# A New Filter Based Cultivation Approach for Improving *Aspergillus* Identification using Matrix-Assisted Laser Desorption/Ionization Time-of-Flight Mass Spectrometry (MALDI-TOF MS)

**DOI:** 10.1007/s11046-021-00603-8

**Published:** 2022-01-10

**Authors:** Husam Salah, Anna Kolecka, Anna Rozaliyani, Retno Wahyuningsih, Saad J. Taj-Aldeen, Teun Boekhout, Jos Houbraken

**Affiliations:** 1 Division of Microbiology, Department of Laboratory Medicine and Pathology, Hamad Medical Corporation, Doha, Qatar; 2 Westerdijk Fungal Biodiversity Institute, Utrecht, The Netherlands; 3 Institute of Biodiversity and Ecosystem Dynamics (IBED), University of Amsterdam, Amsterdam, The Netherlands; 4 Department of Parasitology Faculty of Medicine, Universitas Indonesia, Jakarta, Indonesia; 5 Department of Parasitology Faculty of Medicine, Universitas Kristen Indonesia, Jakarta, Indonesia; 6 University of Babylon, Hilla, Iraq

**Keywords:** MALDI-TOF MS, Clinical filamentous fungi, Identification, Polycarbonate filter, Bruker

## Abstract

Matrix-Assisted Laser Desorption/Ionization Time-of-Flight Mass Spectrometry (MALDI-TOF MS) is widely used in clinical laboratories for routine identification of bacteria and yeasts. However, methodological difficulties are still apparent when applied to filamentous fungi. The liquid cultivation method recommended by Bruker Daltonics GmbH for identification of filamentous fungi by MALDI-TOF MS is labour intensive and time-consuming. In this study, growth of *Aspergillus* species on different (porous) surfaces was investigated with the aim to develop a more reliable, quicker and less laborious identification method using MALDI-TOF MS. Mycelial growth without sporulation mimicking liquid cultivation and reliable MALDI-TOF MS spectra were obtained when *A. fumigatus* strains were grown on and in between a polycarbonate membrane filter on Sabouraud dextrose agar. A database of in-house reference spectra was created by growing *Aspergillus* reference strains (mainly focusing on sections *Fumigati* and *Flavi*) under these selected conditions. A test set of 50 molecularly identified strains grown under different conditions was used to select the best growth condition for identification and to perform an initial validation of the in-house database. Based on these results, the cultivation method on top of a polycarbonate filter proved to be most successful for species identification. This method was therefore selected for the identification of two sets of clinical isolates that mainly consisted of Aspergilli (100 strains originating from Indonesia, 70 isolates from Qatar). The results showed that this cultivation method is reliable for identification of clinically relevant *Aspergillus* species, with 67% and 76% correct identification of strains from Indonesia and Qatar, respectively. In conclusion, cultivation of Aspergilli on top of a polycarbonate filter showed improved results compared to the liquid cultivation protocol recommended by Bruker in terms of percentage of correct identification, ease of MSP creation, time consumption, cost and labour intensity. This method can be reliably applied for identification of clinically important Aspergilli and has potential for identification of other filamentous fungi.

**Supplementary Information:**

The online version contains supplementary material available at 10.1007/s11046-021-00603-8.

## Introduction

Invasive fungal infections (IFI) cause yearly more than 1.5 million deaths worldwide, especially in immunocompromised individuals [1]. They are also associated with considerable healthcare costs [2]. Rapid and accurate identification of the causative fungus (yeast or mould) is crucial for timely initiation of the antifungal therapy and successful treatment [3]. Identification of pathogenic moulds in routine clinical laboratories relies mainly on morphological features (macroscopy and microscopy) and can be challenging [4]. For example, identification may be delayed especially for slow growing moulds that may take up to several weeks of incubation to produce the characteristic morphological features used to determine the genus and species. In addition, rare fungi, particularly cryptic and hybrid species, are emerging as human pathogens [5–7] and are difficult to identify by phenotypic methods alone. These species may exhibit species-specific antifungal susceptibility profiles [8–10] and therefore require an accurate identification for proper patient management. DNA sequencing remains the gold standard for definite identification of pathogenic fungi [11]; however, it is expensive, not commonly available in routine clinical laboratories and requires highly trained personnel.

Matrix-Assisted Laser Desorption/Ionization Time-of-Flight Mass Spectrometry (MALDI-TOF MS) has revolutionised the field of clinical microbiology in the past decade being a quick, easy, inexpensive and accurate method for identification of pathogenic bacteria and fungi [12–15]. The microorganisms are tested either by “direct transfer” of the microorganism to a MALDI-TOF target plate, treatment with formic acid for cell lysis, followed by addition of α-cyano-4-hydroxycinnamic acid (HCCA) matrix solution (extended direct transfer), or by complete protein extraction using ethanol/formic acid according to the Bruker Daltonics GmbH protocol [15, 16]. The first method is generally used for bacteria, the second method is recommended for yeasts and some gram positive bacteria and the third is mostly applied to moulds with prior cultivation in rotating liquid culture medium. Besides sample preparation and the extraction procedure, the accuracy and robustness of identification also depends on the quality of the database used [17].

Due to their biological complexity, variable growth patterns and difficulty of inoculum standardization, the identification of filamentous fungi by MALDI-TOF MS is more complex compared to that of bacteria and yeasts [18]. Bruker Daltonics GmbH recommends a liquid cultivation (LC) method with constant rotation overnight followed by ethanol/formic acid extraction protocol, to produce a uniform growth of fungal mycelia and to minimize the effects of culture conditions on the mass spectrum followed by ethanol/formic acid extraction protocol [19]. In this proposed method, especially processing of the fungal material until extraction is labour-intensive and time consuming. Several methods have been proposed to improve MALDI-TOF MS based identification of filamentous fungi. These included various sample preparation methods, such as mechanical cell lysis [20, 21], cultivation on Sabouraud gentamicin-chloramphenicol agar [22], and by direct analysis of intact fungal spores and/or hyphae [23–25]. Moreover, studies targeting specific mould genera such as *Aspergillus* [23, 25, 26], *Penicillium* [27, 28], *Fusarium* [23, 29–31], and Mucorales [23, 32, 33] were also reported.

In this study (performed during 2014–2016), we investigated a cultivation method for clinical Aspergilli using filters for less laborious and quicker species identification and Main Spectra (MSP) creation using the Bruker MALDI-TOF MS system. The rationale behind the chosen cultivation technique was to avoid or reduce sporulation in order to obtain undifferentiated and uniform mycelia. This could be achieved by growing the Aspergilli on or between surfaces, like membrane filters, on Sabouraud dextrose agar (SDA) [34]. The method was challenged with a set of random test strains in addition to clinical isolates. Some cryptic species complexes were included to further challenge the identification by MALDI-TOF MS. The costs of the analysis, robustness and the accuracy of the identification were important selection criteria during our study.

## Materials and Methods

### Strains

Four sets of strains were used in our study; all were identified using partial calmodulin and/or tubulin gene sequencing as described previously [35]. Cryptic species were included in the in-house databases and the test strains to challenge identification by MALDI-TOF MS. The first set included 55 *Aspergillus* section *Fumigati* strains (strain set 1, Suppl. Table 1) and were obtained from the CBS culture collection (CBS) or the working collection of the Food and Indoor Mycology group (DTO), both housed at the Westerdijk Fungal Biodiversity Institute (WI). The 55 *Aspergillus* section *Fumigati* strains were used to determine the best methodology for generating MSPs. In addition to the section *Fumigati* MSPs, MSPs of another 148 *Aspergillus* strains (Suppl. Table 2) were generated (strain set 2). In this dataset, a large selection of *Aspergillus* section *Flavi* strains was supplemented with species present in the clinical Indonesian and Qatar strain sets (see below, strain set 4). The third set included 50 test strains from the CBS and DTO culture collections (strain set 3, Suppl. Table 3) and these strains were used to determine the best cultivation method for identification and perform an initial validation of the (in-house) databases. The fourth set comprised clinical isolates (mainly Aspergilli) from Indonesia (n = 100) and Qatar (n = 70) (strain set 4, Suppl. Tables 4, 5). These isolates were used to determine the identification success rate of the newly proposed cultivation method in combination with different commercial and in-house databases.

### Culture Conditions

Of each strain, 1–2 µL of spore suspension, which was maintained in 30% glycerol at -80 °C, was transferred to a malt extract agar (MEA, Oxoid) plate and incubated at 25 °C for 48 to 72 h. In order to determine the most optimal cultivation method, small pieces of fungal material were transferred and grown under five different conditions (all on SDA; Difco): 1) directly on the agar surface (control method), 2) on a filter, 3) under a filter, 4) between two filters and 5) in Sabouraud broth (Difco) with constant rotation (recommended method by Bruker Daltonics GmbH). Growth under, between and on four different filters/surfaces were studied: cellophane (CEL) filters (Thermo Fisher Scientific), Parafilm® M Laboratory Film (Bemis Company Inc.), polycarbonate membrane (PC) filters (Maine Manufacturing, LLC; pore size 0.1 μm, diameter 25 mm and 72 mm (the 72 mm was cut in 4 parts)) and mixed ester cellulose filters (Millipore; pore size 0.45 μm). All filters were applied directly on the agar surface, without any pre-treatment. The strains were grown for 48 h at 25 °C prior analysis. Initially, a subset of ten *A. fumigatus* strains was studied under all conditions mentioned above and the results of this initial study were scored based on the degree of growth and sporulation. The four best-scoring conditions (between and on CEL and PC filters) were selected and MSPs of 55 *Aspergillus* section *Fumigati* strains (Suppl. Table 1) were created under these conditions. Strains that failed for MSP creation the first time were repeated once again.

### Protein Extraction and Construction of MSP In-House Database

Ethanol/formic acid extraction was performed according to the Bruker’s protocol [36, 37]. MSPs were created by measuring 24 spots (8 × 3) of each strain. Raw spectra were analysed using Bruker flexAnalysis version 3.3.75.0 software (Bruker Daltonics GmbH, Germany). Out of the 24 spectra, a minimum of 17–20 spectra were selected, after dismissing the ones with low quality, for creating an MSP for each strain. An in-house MSP database was created for strains that were cultivated by each of the methods mentioned previously.

The quality of the in-house section *Fumigati* database was assessed by creation of dendrograms, using the built-in dendrogram clustering package in the Bruker flex Analysis version 3.3.75.0 software, to look for proper species and strain clustering. Besides the MSPs of the selected 55 section *Fumigati* strains (strain set 1), additional MSPs of 148 *Aspergillus* strains grown between and on a PC filter were created (strain set 2).

### Comparison of Cultivation Methods and Initial Validation of In-House MSP Databases

Four different cultivation methods (growth on and between a PC filter, LC and on SDA) were compared using a test set of 50 molecularly characterised strains from the CBS and DTO collections (strain set 3)**.** After growth, the strains of this test set were extracted, spotted twice, analysed and the obtained results were used to validate three databases (the newly created in-house *Aspergillus* section *Fumigati* database, the Bruker filamentous fungi library 1.0 and a combination of both databases). We selected strains other than the ones used for construction of the in-house section *Fumigati* database.

Identification by MALDI-TOF MS was considered correct if the best match (with the highest score) of both spots (if available) was concordant with the identification by DNA sequencing. Identification errors were arbitrarily classified as minor or major based on internal transcribed spacer (ITS) sequence similarity percentage. A MALDI-TOF MS identification was considered a minor error if the ITS similarity between the MALDI-TOF MS and the molecular identification was ≥ 99%. An ITS similarity < 99% was considered as a major identification error.

### Identification of Clinical Isolates and Clinical Validation of Databases

To further test the best cultivation method for identification (growth on top of a PC filter), two sets of clinical isolates from Indonesia (n = 100) and Qatar (n = 70) were identified in duplicate by MALDI-TOF MS using Bruker MicroFlex. These two datasets, and test set 3, were generated by three different individuals.

## Results

### Determination of the Best Culture Conditions

MSPs were generated from section *Fumigati* strains grown under different conditions and using different filters. Growth without filter (directly on SDA) and the LC method recommended by Bruker were used as controls. Initially, a small subset of *A. fumigatus* strains (n = 10) was studied in all possible combinations. No growth was observed for the strains inoculated with Parafilm (under, on and in between) and this method was therefore excluded in the further experiments. The strains grown on SDA with mixed ester cellulose filters showed good growth and sporulation. Our aim was to grow the strains in an undifferentiated state without sporulation and this method was therefore not addressed further. The strains grown directly on SDA (without filter) as well as under filters had no or poor results when measured by the MALDI-TOF MS (data not shown) and these cultivation methods were also excluded. Cultivation of the *A. fumigatus* strains either on top or in between a cellophane or polycarbonate filters resulted in a uniform thin layer of mycelium without or with sparse sporulation. MSPs of 55 *Aspergillus* section *Fumigati* strains were created when grown under these four growth conditions, in addition to the control method using Sabouraud broth. The results of the number of successfully created section *Fumigati* MSPs are summarised in Table 1 and the polycarbonate cultivation method is illustrated in Suppl. Figure 1.

**Table 1:** Growth patterns for *Aspergillus* section *Fumigati* strains and number of successful MSPs obtained by each cultivation method

Cultivation method	Growth pattern	Successful MSPs/Total MSPs (%)
1CEL	Mycelium with good sporulation	43/55 (78%)
2CEL	Mycelial monolayer with sparse sporulation in the middle	36/55 (65%)
1PC	Mycelial monolayer, sparse sporulation	52/55 (94%)
2PC	Mycelial monolayer, sporulation absent	55/55 (100%)
LC	Mycelium, sporulation absent	47/55 (85%)

1CEL: cultivation on top of cellophane filter, 2CEL: cultivation between two cellophane filters, 1PC: cultivation on top of polycarbonate filter, 2PC: cultivation between two polycarbonate filters, LC: liquid cultivation

MSPs were created for strains cultivated on/between cellophane and polycarbonate filters and using the liquid cultivation method. The highest percentage of successful MSPs were generated for strains grown between (100%) and on top (94%) of a polycarbonate filter, followed by the liquid growth condition (84%) and growth on top (78%) and between (65%) a cellophane filter. With the two methods using polycarbonate filters, more MSPs were successfully created with less repeats needed compared to the LC method as recommended by Bruker. The cultivation method using the cellophane filter had a lower success rate for creating of MSPs compared the polycarbonate filter methods. In addition, the time needed to measure MSPs was longer: approx. 180 min were needed to process 50 strains using cellophane filters, while approximately 90–120 min were needed for strains grown on/between polycarbonate filters. Approximately 150 min were needed to measure 50 strains with the liquid method. Furthermore, the cellophane membrane was technically more difficult to work with due to drying of the membrane. Based on these results, the growth conditions using a cellophane filter were excluded in further experiments.

### Quality of the Newly Created *Aspergillus* section *Fumigati* MSP Databases

The quality of the *Aspergillus* section *Fumigati* in-house MSP database was investigated by cluster analysis using the built-in dendrogram application of the Bruker’s BioTyper 3.0 software. The strain clustering was mostly correct for the MSPs made by the 1PC (growth on top of a polycarbonate filter) method (43/52; 83%), though various strains (e.g. *Aspergillus udagawae* DTO 159-C8, *Aspergillus hiratsukae* DTO 017-A3 and *Aspergillus fumigatiaffinis* DTO 203-E3) clustered incorrectly (Suppl. Figure 2). The majority of strains also clustered correctly when grown between two polycarbonate filters (2PC method) (46/55, 84%) (Suppl. Figure 3). In contrast, mainly the *A. fumigatus* strains clustered correctly for MSPs made with the LC in Sabouraud broth (LC) method; most of the other strains showed incorrect clustering.

### Comparison of Cultivation Method and Initial Validation of MSP Databases

A test set of 50 molecularly identified strains (47 *Aspergillus* strains representing 15 species, two *Penicillium* and one *Talaromyces* species) (Suppl. Table 3) was used for comparison of the cultivation methods to select the best method for identification and to validate the newly created MSP database with *Aspergillus* section *Fumigati* strains. Based on the afore mentioned cultivation methods that generated the highest number of successfully created MSPs, cultivation of the test strains on top (1PC method) and between (2PC method) a polycarbonate filter was selected, in addition to the liquid cultivation method and direct inoculation on SDA as controls. The test strains were identified against the newly created in-house database, the Bruker filamentous fungi library 1.0 and a combination of both databases. From the 50 test strains, only the ones present in each database were used for validation, i.e. 40/50, 28/50 and 44/50 for the in-house database, Bruker filamentous fungi library 1.0 (BDAL) and the combination of both databases, respectively. The test strains that were not present in the database were expected not to match with any MSP and should score lower than < 1.700 (no reliable identification). This result can be considered as a valid result. The overall results of the identification and initial validation of the databases are presented in Table 2. The MALDI-TOF MS identification results of the 50 test strains are shown in Suppl. Table 4–6 and details of the minor and major errors are given in Suppl. Tables 7 and 8.

**Table 2:** Initial validation results using 50 *Aspergillus* section *Fumigati* test strains

Databases	In-house (40/10)				BDAL (28/22)				All databases (44/6)			
Cultivation method	Control (SDA)	1PC	2PC	LC	Control (SDA)	1PC	2PC	LC	Control (SDA)	1PC	2PC	LC
***Species included in the database***												
Correct ID (> 2.000)	16 (40%)	37 (93%)	35 (88%)	24 (60%)	4 (14%)	24 (86%)	19 (68%)	18 (64%)	14 (32%)	39 (89%)	37 (84%)	27 (61%)
Correct ID (> 1.700)	17 (43%)	38 (95%)	35 (88%)	26 (65%)	10 (36%)	26 (93%)	24 (86%)	20 (71%)	18 (41%)	40 (91%)	37 (84%)	30 (68%)
No reliable ID	0	0	0	0	0	1 (4%)	0	2 (7%)	2 (5%)	2 (5%)	2 (5%)	1 (2%)
No peaks found	21 (53%)	1 (3%)	4 (10%)	9 (23%)	18 (64%)	1 (4%)	4 (14%)	6 (21%)	23 (52%)	1 (2%)	5 (11%)	9 (20%)
Minor errors	0	0	0	4 (10%)	0	0	0	0	0	1 (2%)	0	2 (5%)
Major errors	2 (5%)	1 (3%)	1 (3%)	1 (3%)	0	0	0	0	1 (2%)	0	0	2 (5%)
***Species not included in the database***												
No peaks found	8 (80%)	0	2 (20%)	0	11 (50%)	0	2 (9%)	3 (14%)	6 (100%)	0	1 (17%)	0
No reliable ID*	2 (20%)	8 (80%)	6 (60%)	8 (80%)	11 (50%)	11 (50%)	16 (73%)	7 (32%)	0	1 (17%)	1 (17%)	1 (17%)
Minor errors	0	2 (20%)	2 (20%)	2 (20%)	0	1 (5%)	1 (5%)	1 (5%)	0	3 (50%)	3 (50%)	3 (50%)
Major errors	0	0	0	0	0	10 (45%)	3 (14%)	11 (50%)	0	2 (33%)	1 (17%)	2 (33%)
***Overview miscellaneous data***												
Total no. of spots with no peaks found	70 (70%)	9 (9%)	21 (21%)	33 (33%)	70 (70%)	9 (9%)	21 (21%)	33 (33%)	70 (70%)	9 (9%)	21 (21%)	33 (33%)
No peaks found (in both spots)	29 (58%)	1 (2%)	6 (12%)	9 (18%)	29 (58%)	1 (2%)	6 (12%)	9 (18%)	29 (58%)	1 (2%)	6 (12%)	9 (18%)
% correct ID (incl. no reliable ID*) (correct / total)	86% (18/21)	92% (45/49)	93% (41/44)	78% (32/41)	71% (15/21)	71% (35/49)	80% (35/44)	61% (25/41)	67% (14/21)	82% (40/49)	86% (38/44)	68% (28/41)
Average % correct ID per database	87%				70%				76%			

^*^A “no reliable ID” is expected for species not included in the database and can therefore be considered as correct. 1PC: growth on top of a polycarbonate filter, 2PC: growth between two polycarbonate filters, LC: liquid cultivation

No interpretable protein profiles (no peaks found) for both spots were obtained in 58% (29/50) of the test strains grown directly on SDA (control method). Higher success rates were measured for the other three cultivation methods. The 1PC method scored the best (2% no peaks found), followed by the 2PC (12% no peaks found) and the LC method (18% no peaks found). A similar result was observed when single spots were compared: 70% of the spots failed for the direct growth method, 9% for the 1PC, 21% for the 2PC and 33% for the LC method (Table 2). The same extraction protocol was used in all analyses. These data therefore show that the percentage of correctly identified strains largely depended on the cultivation method and subsequent harvesting on fungal material. The 1PC method was the most successful for generating mass spectra. Moreover, the LC method was more time consuming (i.e. six and half hours for processing 50 isolates using LC method compared to about two hours for the PC methods).

The identification success rate also depended on the database used. On average, the percentage of correctly identified strains (including the unreliable identifications for the species not present in the database and excluding the strains without mass spectra) was 87%, 70% and 76% for the in-house database, BDAL and a combination of both databases, respectively. The 2PC method scored best in all databases (in-house, 93%; BDAL, 80%; combined, 86%), directly followed by the 1PC method (in-house, 92%; BDAL, 71%; combined, 82%). The percentage of correctly identified strains in the in-house database was lowest for the LC method (78%) and a similar result was obtained for the BDAL database (61%). The strains examined when grown directly on SDA scored lowest in the combined databases (67%), closely followed by the LC method (68%) (Table 2). In summary, the database did not have a large influence on the percentage of correctly identified strains using the 1PC and 2PC method. Interestingly, the number of false positive identifications (Table 2, Suppl. Table 8) in the BDAL database was higher for the 1PC method (11 strains, scores between 1.728–1.885) than those for the 2PC method (4 strains, 1.714–1.912).

Table 3 shows the percentage of MSP matches for the correctly identified test strains. For the test strains that were identified against the in-house database, the strains grown directly on SDA (without filter) and using the 1PC and 2PC methods matched best with MSPs generated using polycarbonate filters (88%, 66%, and 48%, respectively). In contrast, the majority of strains cultivated by LC matched best with 1CEL (35%) and LC (35%) MSPs. When identification protein profiles were compared against all the databases (in-house and BDAL), strains grown directly on SDA and using the 1PC method matched mostly with MSPs from 1PC (72% and 53%, respectively), whereas the strains cultured by LC had the highest match with BDAL MSPs (37%).

**Table 3:** Number of MSP matches for the correctly identified test strains used for the initial validation

MSP match	Cultivation method			
	SDA (Control)	1PC	2PC	LC
*In-house*				
SDA (Control)	1 (6%)	0	0	0
1PC	**15 (88%)**	**25 (66%)**	16 (46%)	4 (15%)
2PC	0	7 (18%)	**17 (48%)**	2 (8%)
1CEL	1 (6%)	4 (10%)	0	**9 (35%)**
2CEL	0	1 (3%)	2 (6%)	2 (8%)
LC	0	1 (3%)	0	**9 (35%)**
*All databases*				
SDA (Control)	2 (11%)	0	2 (5%)	0
1PC	**13 (72%)**	**21 (53%)**	9 (24%)	1 (3%)
2PC	2 (11%)	4 (10%)	16 **(43%)**	6 (20%)
1CEL	1 (6%)	6 (15%)	2 (5%)	3 (10%)
2CEL	0	0	4 (11%)	3 (10%)
LC	0	0	0	6 (20%)
BDAL	0	9 (22%)	4 (11%)	**11 (37%)**

1CEL: growth on a cellophane filter, 2CEL: growth between two cellophane filters, 1PC: growth on a polycarbonate filter, 2PC: growth between two polycarbonate filters, LC: Liquid cultivation.

Bold text: Highest number of MSP matches

### Identification of clinical isolates and clinical validation of databases

#### Indonesian Isolates

Based on the results of the comparison of the various cultivation methods reported above, the 1PC method was selected to identify the clinical isolates from Indonesia and Qatar. The Indonesian test set contained 100 isolates covering 11 species (Suppl. Table 9). These strains were identified against the in-house databases (LC, 1PC and 2PC method) and a combination of these supplemented Bruker’s filamentous fungi library 1.0 (collectively including 674 MSPs). The overall identification results using the in-house and combined databases are summarised in Table 4.

**Table 4:** Validation of the in-house and BDAL databases using Indonesian clinical isolates

	Percentage of total number of investigated strains (n = 100)			
	All (%)	LC (%)	1PC (%)	2PC (%)
Correctly identified (score ≥ 2.000)	67	43	60	53
Correctly identified (score ≥ 1.700)	67	45	63	56
Minor errors	29	41	25	31
Major errors	2	12	2	3
No reliable identification	1	1	9	9
No peaks found	1	1	1	1

1PC: Database of strains grown on a polycarbonate filter, 2PC: Database of strains grown between two polycarbonate filters

LC: Database of strains grown in liquid culture, All: Combination of all databases

No interpretable protein profiles were obtained in 15 of the 200 analysed spots (7.5%) and both spots failed for one *A. fumigatus* isolate (1%). The percentage of correctly identified isolates depended on the database used and were 43% (LC), 53% (2PC), 60% (1PC) and 67% (combined databases). Nine isolates (9%) representing five species had “no reliable identification” since they were not present in the 1PC and 2PC databases, and isolate DTO 310-G1 (*Aspergilus aculeatinus*) could not be reliably identified using all databases. Due to the absence of these species in the databases, these results can be considered correct. Furthermore, the percentage of major and minor identification errors of closely related species was highest in the LC database, 12 and 41% respectively, and lowest in the 1PC database (2 and 25%, respectively). Most of the major identification errors with the LC database were for members of *Aspergillus* section *Flavi*, namely *A. flavus* (n = 3) and *A. tamarii* (n = 7), in addition to two isolates of *A. calidoustus*, a species not present in the databases and identified as *A. ustus* (ITS similarity = 93%). Identification against the 1PC and 2PC databases resulted in two major errors with two *A. fumigatus* isolates that were identified as *A. nishimurae* (ITS similarity = 97.8%). In addition, identification against the 2PC database had another major identification error with an *A. flavus* isolate that was identified as *A. sojae* (ITS similarity = 96.6%).

**Fig. 1:**
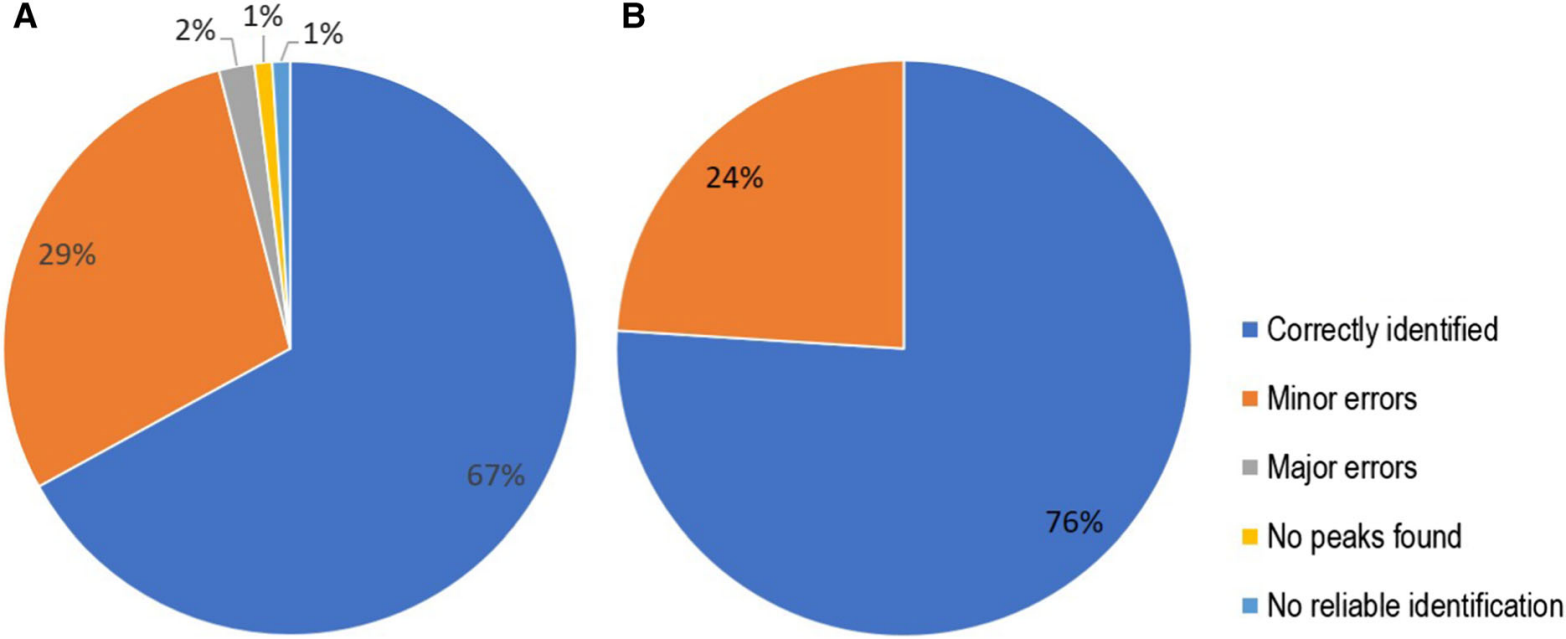
**a** Identification results of Indonesian clinical isolates against combined databases (in-house and BDAL) **b** Identification results of Qatar clinical isolates against all databases (in-house and BDAL)

When comparing against all MSP databases, 67% of the Indonesian clinical isolates were correctly identified, 29% showed minor errors (cryptic species), 2% major errors (two *A. calidoustus* isolates were identified as *A. ustus*), 1% had no reliable identification and no protein profiles could be created for one isolate (1%) (Fig. 1a). If we consider the minor errors correct (≥ 99% ITS similarity), then the percentage would increase to 96%. The identified isolates matched with MSPs from the 1PC (n = 48), 2PC (n = 28) and BDAL (n = 20) databases.

#### Qatar Isolates

Like the Indonesian isolates, the set of clinical isolates from Qatar was grown using the 1PC method. All spectra were identified using the combined database (in-house and BDAL). This set consisted of 70 clinical *Aspergillus* isolates comprising 14 species (Suppl. Table 10). All isolates were identified with scores > 2.000. The spectra of the identified isolates matched with MSPs from BDAL (n = 31, 44%), 1PC (n = 26, 37%), 2PC (n = 4, 6%), LC (n = 3, 4%), 2CEL (n = 6, 9%) databases. Out of 70 isolates, 53 (76%) were correctly identified and 17 (24%) minor identification errors were encountered, with no major identification errors (Fig. 1b). From the 17 isolates with identification errors, seven *A. welwitschiae* isolates (section *Nigri*) were identified as *A. niger*, three *A. citrinoterreus* isolates (section *Terrei*) as *A. terreus*, five *A. flavus* (section *Flavi*) as *Aspergillus aflatoxiformans* or *Aspergillus pipericola* and two isolates from section *Nidulantes* (*A. quadrilineatus* and *A. sublatus*) were both identified as *A. nidulans* (see Suppl. Table 11).

Of the correctly identified strains, 29/53 (55%) matched with MSPs from the BDAL database. Seventeen of these were *A. terreus* and two *A. nidulans*. All correctly identified *Aspergillus* section *Flavi* isolates (n = 16) matched with MSPs from 1PC (n = 13) and 2PC databases (n = 3). Three isolates of *Aspergillus* section *Nigri* matched with MSPs from the 1PC database and one isolate each from *Aspergillus* sections *Nidulantes* and *Aspergillus*, matched with MSPs from the 2CEL database. Nine *A. fumigatus* isolates had best matches with BDAL and three with the in-house LC database.

### Time and Expenditure

On average, the preparation time from harvesting the mycelia until the protein extraction step was 130 min for 50 strains using the 1PC and 2PC cultivation methods and 400 min for the LC method; hence the labour cost for the liquid cultivation is approximately three times higher than for the 1PC and 2PC cultivation method. Compared to the 1PC method, the cost of the consumables (filters, SDA plates/broth) was approx. 60% higher for the 2PC and LC method. In conclusion, the 1PC method performed best in respect to time and costs, followed by the 2PC cultivation method which was more expensive due to the use to two filters (same processing time needed). The LC method performed worst in costs due to the longer processing time needed.

## Discussion

### Newly Developed Cultivation Method

The current study aimed at improving the identification of filamentous fungi by Bruker’s MALDI-TOF MS system using an alternative cultivation method to the LC method recommended by Bruker Daltonics GmbH. We tested several cultivation methods with various filters/surfaces placed on SDA. The 1PC method showed to be the best in terms of robustness of identification, labour intensity and cost. This method could be reliably applied for the identification of clinically significant Aspergilli and has potential for identification of other filamentous fungi.

An in-house MSP database was constructed using *Aspergillus* section *Fumigati* strains that were cultivated on or between various surfaces and filters placed on SDA, in addition to LC and cultivation on SDA without filters (control). The in-house databases were initially validated using a test set of 50 strains and subsequently expanded with *Aspergillus* section *Flavi* species and other taxa occurring in the sets of clinical isolates from Indonesia and Qatar. In the current study, we did not aim for constructing a broad in-house database. For the purpose of this study, we made a limited database of *Aspergillus* species to evaluate the alternative cultivation methods other than LC recommended by Bruker Daltonics GmbH. The current study showed that cultivation on a polycarbonate filter is a reliable method for identification of clinically relevant *Aspergillus* species with 67% and 76% correct identifications for the clinical sets of strains from Indonesia and Qatar, respectively. When minor errors are counted as correct, the rate would rise to 96% for the Indonesian and 100% of the Qatari clinical isolates. The isolates with minor identification errors were mostly identified as phylogenetically closely related species as reported previously [38]. These strains had ITS sequence similarity of ≥ 99% and could not be differentiated by MALDI-TOF MS probably due to high similarities in their protein spectra. This can lead to misidentification of closely related species as reported in previous studies [26, 39–41]. Some species were not present in the in-house and/or the Bruker commercial databases and therefore either not identified (score < 1.700) or identified as a closely related species. Few isolates were incorrectly identified and were considered major identification errors (ITS sequence similarity < 99%). Inclusion of more closely related, well-characterized species in the databases could reduce these identification errors [38].

*Aspergillus* colonies can consist of various structures, like mycelium, conidiophores with conidia and ascomata containing asci and ascospores. Both intrinsic (e.g. nutrients, pH, water activity) and extrinsic factors (e.g. oxygen, temperature) influence the colony morphology [42]. These different colony morphologies can impede the acquisition of reproducible spectra. L'Ollivier et al. [43] have demonstrated that fungal material harvested at different incubation times from the same colony yielded different spectra. Several attempts were made by researchers to improve MALDI-TOF MS based identification of filamentous fungi. The majority focused on sample preparation and the protein extraction steps, and a limited number of investigations studied growth conditions [21–23, 44–46]. In this study we aimed for mycelial growth without sporulation mimicking liquid cultivation. We showed that growth conditions (and subsequent harvesting of fungal material) have a large impact on the success rate of generating interpretable protein profiles, and therefore also directly influencing the identification success. Growth on a polycarbonate filter outperformed the other tested growth conditions: no interpretable protein profiles were obtained in 70% of the spots for the direct growth method, followed by 33% for the LC method, 21% for the 2PC method and 9% for the 1PC method. This low percentage of interpretable protein profiles using the 1PC method was also observed when studying the Indonesian and Qatar isolates. These analyses were performed by three different individuals, showing the robustness of the method.

A similar technique using a permeable membrane on an agar surface is recently commercialized and is on the market under the name ID Fungi Plate (Conidia, Quincieux, France). The use of this filter-based method was found to be more easy for harvesting fungal material and results in a more rapid identification of moulds than conventional methods [45, 46]. These data underlines the potential of the use of filters for the identification of moulds using MALDI-TOF MS. In this manuscript we provide a non-commercial, easy to use, and home-made version of a cultivation technique using polycarbonate filter that can be applied in virtually all labs using MALDI-TOF MS for identification of filamentous fungi.

### Identification of Clinical Strains

When examining the identification results of the Indonesian clinical strains, the databases created using the 1PC and 2PC method performed better than that with MSPs generated using the LC method. Moreover, the LC method had the highest percentage of major errors (10%) compared to 1PC (2%) and 2PC (3%) methods. Though these results might be biased since the Indonesian clinical strains were cultivated only with the 1PC method, identification against all MSP databases using the 1PC cultivation showed a comparable correct identification rate. Most of the identified isolates matched with 1PC database (48%) followed by 2PC (28%) and BDAL (20%). These results indicate a slight deviation in the spectral peaks acquired with different cultivation methods which was also highlighted by Cassagne et al. [22]. Twelve major identification errors were associated with identification against the LC database. Most of these major identification errors using the LC database were for *Aspergillus* section *Flavi* members, namely *A. flavus* (n = 3) and *A. tamarii* (n = 7). *Aspergillus* section *Flavi* species are difficult to distinguish using phenotypic methods and some species are phylogenetically very closely related (e.g. *A. flavus*, *A. aflatoxiformans, A. austwickii*, *A. cerealis*, *A. pipericola*) [47]. Identification of these species by MALDI-TOF MS proved to be difficult in our study. In a previous study by Hedayati et al., identification by MALDI-TOF MS could not separate members of *Aspergillus* section *Flavi* using Bruker’s commercial database [48]. In contrast, Quéro et al. evaluated the applicability of MALDI-TOF MS to identify 68 strains that belong to 23 species of *Aspergillus* section *Flavi* using the VITEK MS system (bioMérieux, Marcy l’Etoile, France) [49]. The spectra of 16 species were integrated into the bioMérieux spectral database and identification performances were assessed by cross-validation. Interestingly, more than 99% of the spectra were assigned to the correct species. For example, they were able to discriminate between *A. parasiticus* and *A. novoparasiticus* that are very closely related and share the same partial beta-tubulin gene sequence [47] and were also able to distinguish between aflatoxin producing section *Flavi* species (*A. aflatoxiformans*, *A. arachidicola*, *A. flavus*, *A. luteovirescens*, *A. minisclerotigenes*, *A. nomius*, *A. novoparasiticus*, *A. parasiticus*, *A. pseudotamarii*, *A. sergii*, *A. transmontanensis*) from non-producers (*A. avenaceus*, *A. caelatus*, *A. leporis*, *A. sojae*, *A. tamarii*). It should be noted that the cross validation of their in-house database was performed using the same set of strains that was also used to build the database [49]. In contrast, we used different sets of strains in our study for database construction, initial validation and identification of clinical isolates. Furthermore, we included also species that were not present in the database that served as negative controls.

Minor identification errors due to the presence of closely related species were also observed for clinical isolates belonging to *Aspergillus* section *Nigri*. Isolates of *A. welwitschiae* (section *Nigri*) were mostly identified as *A. niger* and *A. neoniger* as *A. tubingensis*. *Aspergillus welwitschiae* and *A. neoniger* were not present in all databases and were therefore identified as another closely related species (*A. niger* or *A. tubingensis*). In contrast, all isolates of *A. tubingensis* (section *Nigri*) were correctly identified. Similarly, all *A. terreus* isolates were correctly identified whereas *A. citrinoterreus* isolates were identified as *A. terreus* (minor error), indicating that these phylogenetically closely related species cannot be distinguished by MALDI-TOF MS due to their highly similar protein profiles. Similar results were reported in various studies. Vidal-Acuña et al. reported that among 179 clinical Aspergilli grown on solid medium (Sabouraud-chloramphenicol agar plates), *A. tubingensis* was the only cryptic species identified by MALDI-TOF MS using an in-house database of clinical and reference *Aspergillus* strains including cryptic species grown in liquid medium [50]. Tsang et al. could not distinguish *A. niger*, *A. tubingensis* and *A. welwitschiae* using the LC method as recommended by Bruker [51] and Young Yoo et al., using the MicroIDSys Elite (ASTA Corp., South Korea) MALDI-TOF MS system, could not distinguish *A. niger* and *A. tubingensis* [52]. However, in contrast to our results, D’hooge et al. were able to differentiate *A. niger*, *A. tubingensis*, *A. welwitschiae, A. brasiliensis*, *A. brunneoviolaceus* and *A. neoniger* [53].

Clinically, detailed antifungal susceptibility patterns of cryptic *Aspergillus* species are not well investigated. Gonçalves et al. showed that *A. tamarii* was less susceptible to triazoles compared to other members of section *Flavi* [54]. In other studies by Alcazar-Fuoli et al. and Vermeulen et al., *A. tubingensis* was found to be more resistant to various antifungal drugs compared to other members of section *Nigri* [55, 56]. Imbert et al. reported variable susceptibility patterns for amphotericin B among species of *Aspergillus* section *Terrei* [57]. In another recent study, Imbert et al. also observed high azoles MICs for cryptic species of *Aspergillus* section *Fumigati*, except for *A. hiratsukae* and *A. tsurutae*, and high MICs for amphotericin B were obtained for *A. lentulus* and *A. udagawae* [58]. Moreover, a recent study of 112 *Aspergillus* section *Nigri* isolates by Carrara et al. showed that *A. tubingensis* exhibited higher azoles MICs compared to *A. welwitschiae* [59]. More research on the susceptibility to antifungal agents with larger numbers of cryptic species will result in more insight in their antifungal susceptibility profiles; however, quick and reliable identification tools are also needed. Our data show that strains of closely related species are sometimes difficult to distinguish by MALDI-TOF MS which is partly also due to incomplete MSP databases. This is in agreement with previous studies that showed improved reliability of MALDI-TOF MS identification by constructing an in-house database of strains that were previously accurately identified by DNA sequencing [22, 37, 60–65].

## Conclusion

In conclusion, this study showed that cultivation on top of a polycarbonate membrane filter is a useful method for in-house MSP database construction as well as for reliable identification of *Aspergillus* species by MALDI-TOF MS. Our results showed that this method surpasses the liquid cultivation technique proposed by Bruker Daltonics GmbH in terms of processing time of the sample (e.g. harvesting mycelium) and robustness of identification. It can be reliably applied to routine clinical laboratories for fast *Aspergillus* identification and has potential for other clinically relevant moulds as well. Taxonomic issues remain with the identification of the closely related species, which is also observed when using phenotype-based identification schemes or ITS sequencing. Constructing an in-house MSP database with more genera/species of the rare and emerging pathogenic filamentous fungi would further enhance the efficiency of their identification by MALDI-TOF MS. In order to reduce misidentifications, as well as to increase the likelihood to correctly identify sibling species, it is highly important to use only highly reliably identified strains/isolates for construction of the MSP reference databases.

## Supplementary Information

Below is the link to the electronic supplementary material.

Supplementary file1 (DOCX 8466 kb)

Supplementary file2 (DOCX 197 kb)
