# Supplementary material for: A New Filter Based Cultivation Approach for Improving Aspergillus Identification using Matrix-Assisted Laser Desorption/Ionization Time-of-Flight Mass Spectrometry (MALDI-TOF MS)
Source: Mycopathologia. 2022 Jan 10;187(1):39–52. doi: 10.1007/s11046-021-00603-8 (PMC8807449; doi:10.1007/s11046-021-00603-8)
Supplement: Supplementary file 1 — Supplementary file1 (DOCX 8466 kb) [file 11046_2021_603_MOESM1_ESM.docx]

**A new cultivation approach for improving *Aspergillus* identification using Matrix-Assisted Laser Desorption/Ionization Time-Of-Flight Mass Spectrometry (MALDI-TOF MS)**

Husam Salah^1, 2^, Anna Kolecka^2*^, Anna Rozaliyani^4^, Retno Wahyuningsih^4, 5^, Saad J. Taj-Aldeen^1, 6^, Teun Boekhout^2, 3^ and Jos Houbraken^2^

^1^Division of Microbiology, Department of Laboratory Medicine and Pathology, Hamad Medical Corporation, Doha, Qatar.

^2^ Westerdijk Fungal Biodiversity Institute, Utrecht, The Netherlands.

^3^Institute of Biodiversity and Ecosystem Dynamics (IBED), University of Amsterdam, The Netherlands.

^4^Department of Parasitology Faculty of Medicine, Universitas Indonesia, Jakarta, Indonesia.

^5^Department of Parasitology Faculty of Medicine, Universitas Kristen Indonesia, Jakarta, Indonesia.

^6^University of Babylon, Hilla, Iraq.

*Currently, Orthros Medical B.V.


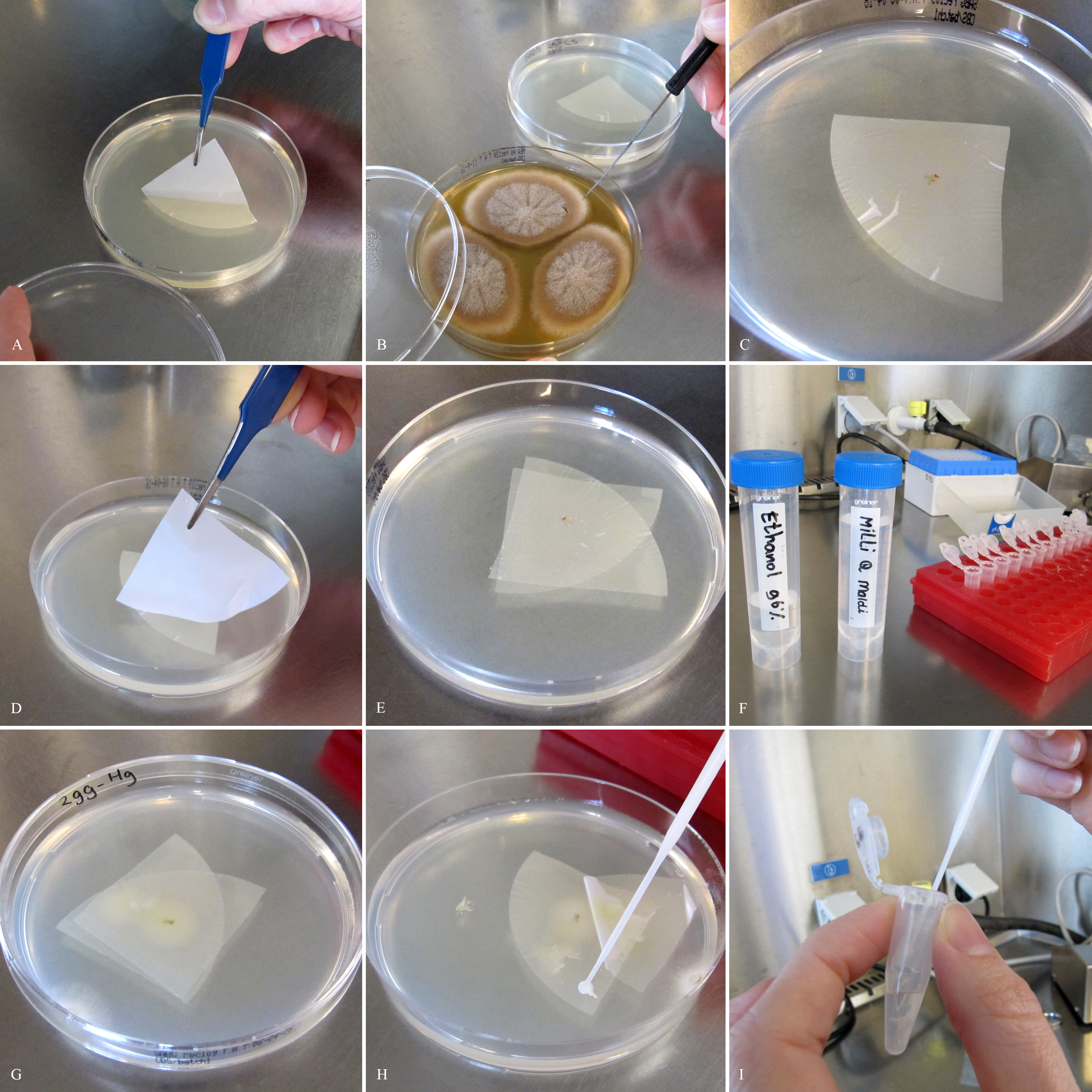


**Supplementary Fig. I.** Illustration of the cultivation method between two polycarbonate (PC) filters. A. Placement of the PC filter on SDA medium. B–C. Inoculation of the PC filter. D–E. Placement of the second PC filter on top of the first PC filter. F. Materials used for the protein extraction. G. Strain grown for 48 h at 25 °C showing thin transparent fungal material (mainly mycelium) between the two filters. H–I. Collection of the fungal material from the PC filter and start of the protein extraction.

**
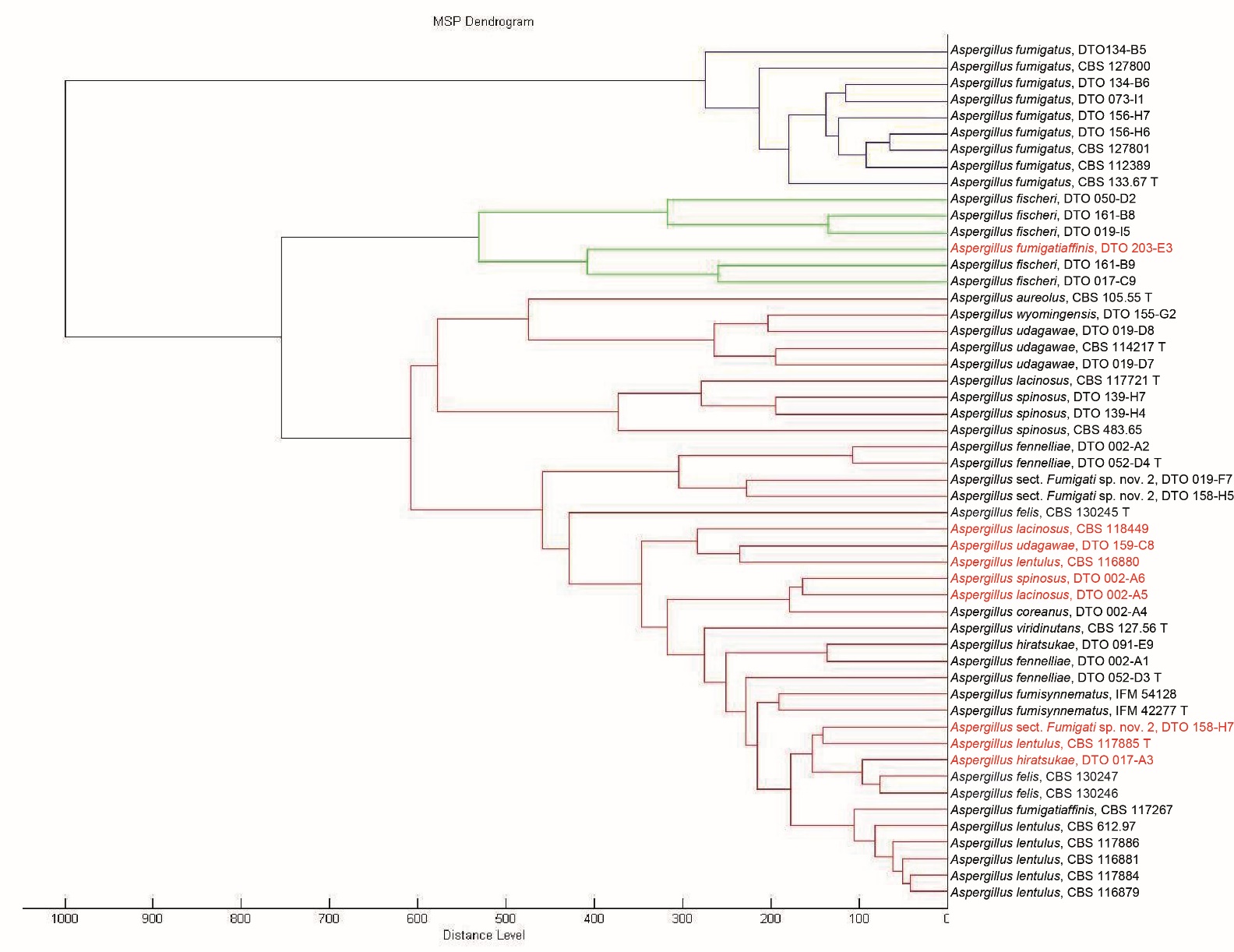
 Supplementary Fig. II.** Dendrogram of the *Aspergillus* section *Fumigati* in-house database based on spectra made from strains grown on top of a polycarbonate filter. 83 % (43/52) of the strains clustered correctly and the wrongly clustered strains are indicated in red.

**
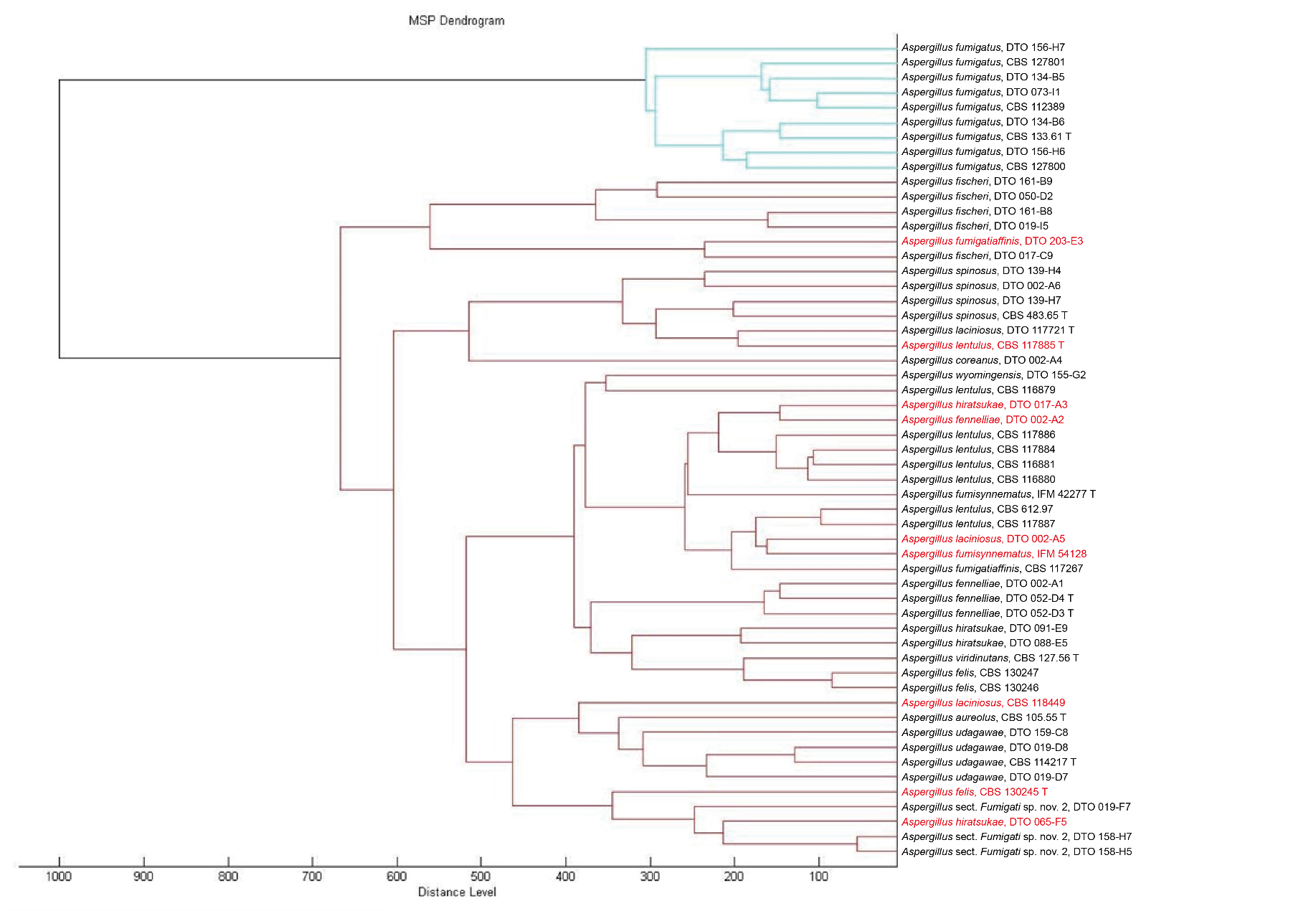
 Supplementary Fig. III.** Dendrogram of *Aspergillus* section *Fumigati* in-house database based on spectra made from strains grown between polycarbonate filters. 84 % (46/55) of the strains clustered correctly and the wrongly clustered strains are indicated in red.
