# Supplementary material for: A New Filter Based Cultivation Approach for Improving Aspergillus Identification using Matrix-Assisted Laser Desorption/Ionization Time-of-Flight Mass Spectrometry (MALDI-TOF MS)
Source: Mycopathologia. 2022 Jan 10;187(1):39–52. doi: 10.1007/s11046-021-00603-8 (PMC8807449; doi:10.1007/s11046-021-00603-8)
Supplement: Supplementary file 2 — Supplementary file2 (DOCX 197 kb) [file 11046_2021_603_MOESM2_ESM.docx]

**A new cultivation approach for improving *Aspergillus* identification using Matrix-Assisted Laser Desorption/Ionization Time-Of-Flight Mass Spectrometry (MALDI-TOF MS)**

Husam Salah^1, 2^, Anna Kolecka^2*^, Anna Rozaliyani^4^, Retno Wahyuningsih^4, 5^, Saad J. Taj-Aldeen^1, 6^, Teun Boekhout^2, 3^ and Jos Houbraken^2^

^1^Division of Microbiology, Department of Laboratory Medicine and Pathology, Hamad Medical Corporation, Doha, Qatar.

^2^ Westerdijk Fungal Biodiversity Institute, Utrecht, The Netherlands.

^3^Institute of Biodiversity and Ecosystem Dynamics (IBED), University of Amsterdam, The Netherlands.

^4^Department of Parasitology Faculty of Medicine, Universitas Indonesia, Jakarta, Indonesia.

^5^Department of Parasitology Faculty of Medicine, Universitas Kristen Indonesia, Jakarta, Indonesia.

^6^University of Babylon, Hilla, Iraq.

*Currently, Orthros Medical B.V.

**Suppl. Table I: *Aspergillus* section *Fumigati* in-house database species list; 55 strains (set 1).**

| Species (number of strains tested) | Strain no. |
| --- | --- |
| *Aspergillus aureolus* (1) | CBS 105.55 T |
| *Aspergillus coreanus* (1) | DTO 002-A4 |
| *Aspergillus felis* (3) | CBS 130246, CBS 130245 T, CBS 130247 |
| *Aspergillus fennelliae* (4) | DTO 002-A1, DTO 002-A2, DTO 052-D3 T, DTO 052-D4 T |
| *Aspergillus fischeri* (5) | DTO 019-I5, DTO 017-C9, CBS 420.96 (= DTO 050-D2), DTO 161-B8, DTO 161-B9 |
| *Aspergillus fumigatiaffinis* (2) | CBS 117267, DTO 203-E3 |
| *Aspergillus fumigatus* (9) | CBS 133.61 T, CBS 112389, CBS 127800, CBS 127801, DTO 134-B5, DTO 134-B6, DTO 156-H6, DTO 156-H7, DTO 073-I1 |
| *Aspergillus fumisynnematus* (2) | CBS 141446 T, DTO 171-B2 |
| *Aspergillus hiratsukae* (4) | DTO 017-A3, DTO 091-E9, DTO 088-E5, DTO 065-F5 |
| *Aspergillus laciniosus* (3) | CBS 117721 T, CBS 118449, CBS 117065 |
| *Aspergillus lentulus* (8) | CBS 116879, CBS 116880, CBS 116881, CBS 117884, CBS 117885 T, CBS 117886, CBS 117887, CBS 612.97 |
| *Aspergillus spinosus* (4) | DTO 002-A6, DTO 139-H4, DTO 139-H7, CBS 483.65 T |
| *Aspergillus udagawae* (4) | CBS 114217 T, DTO 159-C8, DTO 019-D7, DTO 019-D8 |
| *Aspergillus viridinutans* (1) | CBS 127.56 T |
| *Aspergillus wyomingensis* (1) | DTO 155-G2 |
| *Aspergillus* sp. 2 (new species related to *A. lentulus*) (3) | DTO 019-F7, DTO 158-H7, DTO 158-H5 |

T: type strain, CBS: CBS culture collection, DTO: Internal working collection of department of Food and Indoor Mycology (both collection housed at the Westerdijk Institute (WI), Utrecht, the Netherlands).

**Suppl. Table II: Additional strains used for the construction of the in-house clinical *Aspergillus* database; 148 strains (set 2).**

| **Species (number of strains tested)** | **Section** | **Strain no.** |
| --- | --- | --- |
| *Aspergillus chevalieri* (4) | *Aspergillus* | DTO 196-H8, DTO 206-G1, DTO 346-B7, DTO 346-C5 |
| *Aspergillus ochraceus* (5) | *Circumdati* | DTO 048-F2, DTO 050-H1, DTO 077-E4, DTO 203-E2, DTO 298-I5 |
| *Aspergillus pallidofulvus* (4) | *Circumdati* | DTO 021-G2, DTO 080-I7, DTO 293-D4, DTO 304-G8 |
| *Aspergillus aflatoxiformans* (3) | *Flavi* | CBS 133923, CBS 135405, CBS 121.62 |
| *Aspergillus alliaceus* (4) | *Flavi* | CBS 536.65 T, CBS 110.26, CBS 132161, DTO 203-G9, DTO 305-B9 |
| *Aspergillus arachidicola* (4) | *Flavi* | CBS 117610 T, CBS 117612, CBS 117615, DTO 046-A5 T |
| *Aspergillus aspearensis* (3) | *Flavi* | CBS 143672 T, CBS 143673, CBS 143671 |
| *Aspergillus austwickii* (3) | *Flavi* | CBS 143677 T, CBS 135406, CBS 143678 |
| *Aspergillus avenaceus* (4) | *Flavi* | CBS 109.46 T, CBS 109.48, CBS 237.65, CBS 102.45 |
| *Aspergillus bertholletiae* (4) | *Flavi* | DTO 223-D5, DTO 223-D2, DTO 223-D4, DTO 223-D6 |
| *Aspergillus caelatus* (3) | *Flavi* | CBS 763.97, DTO 073-B7, DTO 073-C1 |
| *Aspergillus cerealis* (3) | *Flavi* | DTO 228-F4, DTO 228-F5, DTO 228-F6 |
| *Aspergillus coremiiformis* (2) | *Flavi* | CBS 553.77 T, DTO 034-G7 |
| *Aspergillus flavus* (8) | *Flavi* | CBS 100927 T, DTO 016-I5, CBS 542.69, DTO 281-F5, DTO 281-H9, DTO 300-C7, DTO 252-C6, DTO 281-D9 |
| *Aspergillus lanosus* (2) | *Flavi* | CBS 650.74, DTO 046-B6 |
| *Aspergillus leporis* (4) | *Flavi* | CBS 125914, CBS 151.66 T, CBS 349.81, CBS 129235 |
| *Aspergillus luteovirescens* (4) | *Flavi* | CBS 620.95 T, DTO 073-C2, DTO 073-C3, CBS 117187 |
| *Aspergillus minisclerotigenes* (4) | *Flavi* | DTO 045-F5, DTO 045-F6, DTO 228-H1, CBS 117635 T |
| *Aspergillus mottae* (1) | *Flavi* | CBS 130016 T |
| *Aspergillus nomiae* (4) | *Flavi* | CBS 260.88, DTO 161-F1, DTO 161-F2, CBS 399.93 |
| *Aspergillus novoparasiticus* (3) | *Flavi* | CBS 126849, CBS 126849 T, CBS 126850 |
| *Aspergillus parasiticus* (8) | *Flavi* | CBS 100926 T, DTO 203-H7, DTO 285-G9, CBS 115.37, DTO 318-D2, DTO 261-F8, DTO 203-C4, DTO 173-C3 |
| *Aspergillus pipericola* (1) | *Flavi* | CBS 143680 T |
| *Aspergillus pseudocaelatus* (1) | *Flavi* | CBS 117616 T |
| *Aspergillus pseudonomiae* (4) | *Flavi* | CBS 119388 T, CBS 123902, DTO 177-G7, DTO 303-A4 |
| *Aspergillus pseudotamarii* (1) | *Flavi* | CBS 766.97 T |
| *Aspergillus sojae* (2) | *Flavi* | CBS 100928 T, DTO 173-C3 |
| *Aspergillus tamarii* (4) | *Flavi* | CBS 484.65, DTO 066-A1, CBS 104.13 T, CBS 133097 |
| *Aspergillus togoensis* (1) | *Flavi* | CBS 272.89 T |
| *Aspergillus vandermerwei* (1) | *Flavi* | CBS 132171 |
| *Aspergillus caespitosus* (4) | *Nidulantes* | DTO 053-D1, DTO 053-D3, DTO 060-B6, DTO 325-C1 |
| *Aspergillus nidulans* (5) | *Nidulantes* | DTO 042-C6, DTO 047-H9, DTO 065-F9, DTO 172-I2, DTO 178-B3 |
| *Aspergillus quadrilineatus* (4) | *Nidulantes* | DTO 048-A9, DTO 319-F9, DTO 319-G2, DTO 346-C8 |
| *Aspergillus luchuensis* (6) | *Nigri* | DTO 241-B3, DTO 241-B3, DTO 067-I1, DTO 223-F1, DTO 260-B4, DTO 173-A6 |
| *Aspergillus niger* (9) | *Nigri* | DTO 072-D2, DTO 240-H2, DTO 293-G2, DTO 008-A4, DTO 240-E8, DTO 029-B1, DTO 161-E9, DTO 240-I4, DTO 316-E4 T |
| *Aspergillus tubingensis* (6) | *Nigri* | DTO 260-D4, DTO 241-C8, DTO 299-H8, DTO 252-A8, DTO 178-C3, DTO 312-D5 |
| *Aspergillus welwitschiae* (7) | *Nigri* | DTO 008-C5, DTO 134-I4, DTO 260-H1, DTO 267-I3, DTO 297-B7, DTO 299-H9, DTO 321-E3 |
| *Aspergillus citrinoterreus* (4) | *Terrei* | DTO 032-G4, DTO 141-F4, DTO 224-I5, DTO 331-H6 |
| *Aspergillus terreus* (4) | *Terrei* | DTO 025-C5, DTO 032-G1, DTO 127-I7, DTO 224-G5 |

T: type strain, CBS: CBS culture collection, DTO: Internal working collection of the department of Food and Indoor Mycology, both housed at WI.

**Suppl. Table III: Test strains used for the initial validation of the *Aspergillus* section *Fumigati* in-house database (n=50) (set 3).**

| **Name (number of strains tested)** | **Strain no.** |
| --- | --- |
| *Aspergillus calidoustus* (1) | DTO 057-D8 |
| *Aspergillus felis* (3) | DTO 131-E3 (= CBS 130248), DTO 131-G3, DTO 176-F1 |
| *Aspergillus fischeri* (5) | DTO 010-G1, DTO 017-D1, DTO 019-I7, DTO 027-F8, DTO 164-H5 (= CBS 544.65) |
| *Aspergillus fumigatiaffinis* (1) | DTO 204-F4 |
| *Aspergillus fumigatus* (23) | DTO 010-D2, DTO 012-C5, DTO 028-D7, DTO 041-C8, DTO 064-C3, DTO 073-I1, DTO 075-I1, DTO 076-A1, DTO 082-E4, DTO 084-D5, DTO 085-F8, DTO 086-C1, DTO 086-C4, DTO 091-I5, DTO 094-E3, DTO 094-E4, DTO 109-A9, DTO 124-E2, DTO 150-E9, DTO 152-G6, DTO 167-H1, DTO 244-G3, DTO 271-A3 |
| *Aspergillus fumisynnematus* (2) | DTO 171-A9, DTO 171-B1 |
| *Aspergillus* sp. 1 (new species related to *A. lentulus*) (1) | DTO 001-E4 (= CBS 153.89) |
| *Aspergillus hiratsukae* (1) | DTO 227-I7 |
| *Aspergillus lentulus* (3) | DTO 018-G7 (= CBS 147312), DTO 158-H4 (= CBS 175.97), DTO 179-A2 |
| *Aspergillus niger* (1) | DTO 241-E4 |
| *Aspergillus novofumigatus* (1) | DTO 003-H2 (= CBS 117519) |
| *Aspergillus parasiticus* (1) | DTO 258-D1 |
| *Aspergillus terreus* (1) | DTO 270-H3 (= CBS 136781) |
| *Aspergillus thermomutatus* (1) | DTO 003-E7 (= CBS 138439) |
| *Aspergillus udagawae* (2) | DTO 019-D8, DTO 283-D3 |
| *Penicillium glabrum* (1) | DTO 084-G3 (= CBS 126336) |
| *Penicillium rubens* (1) | DTO 149-A3 (= CBS 132069) |
| *Talaromyces pinophilus* (1) | DTO 442-G2 |

CBS: CBS culture collection, DTO: Internal working collection of department of Food and Indoor Mycology, both housed at WI.

**Suppl. Table IV: Identification results of the 50 test strains (set 3) against in-house *Aspergillus* section *Fumigati* database.**

| **Strain no.** | **Molecular ID** | **SDA (control)** | | | **1PC** | | | **2PC** | | | **LC** | | |
| --- | --- | --- | --- | --- | --- | --- | --- | --- | --- | --- | --- | --- | --- |
|  |  | Spot#1 | Spot#2 | MALDI ID | Spot#1 | Spot#2 | MALDI ID | Spot#1 | Spot#2 | MALDI ID | Spot#1 | Spot#2 | MALDI ID |
| DTO 057-D8 | *Aspergillus calidoustus* | 0 | 0 | No peaks found | 0.811 | 0.911 | No reliable ID | 0 | 0 | No peaks found | 0.918 | 1.223 | No reliable ID |
| DTO 131-G3 | *Aspergillus felis* | 0 | 2.233 | *Aspergillus felis* | 2.549 | 2.584 | *Aspergillus felis* | 2.627 | 2.625 | *Aspergillus felis* | 2.464 | 2.407 | *Aspergillus felis* |
| DTO 176-F1 | *Aspergillus felis* | 1.998 | 0 | *Aspergillus felis* | 2.405 | 2.445 | *Aspergillus felis* | 2.400 | 2.495 | *Aspergillus felis* | 1.975 | 2.482 | *Aspergillus felis* |
| DTO 131-E3 | *Aspergillus felis* (contaminated with *A. fumigatus*) | 2.470 | 2.118 | *Aspergillus fumigatus* | 2.634 | 2.638 | *Aspergillus fumigatus* | 2.598 | 2.543 | *Aspergillus fumigatus* | 2.435 | 2.413 | *Aspergillus fumigatus* |
| DTO 010-G1 | *Aspergillus fischeri* | 2.412 | 2.214 | *Aspergillus fischeri* | 2.547 | 2.631 | *Aspergillus fischeri* | 2.631 | 2.702 | *Aspergillus fischeri* | 0 | 2.350 | *Aspergillus lentulus* |
| DTO 017-D1 | *Aspergillus fischeri* | 2.382 | 2.333 | *Aspergillus fischeri* | 2.678 | 2.659 | *Aspergillus fischeri* | 2.688 | 2.721 | *Aspergillus fischeri* | 2.395 | 2.480 | *Aspergillus fischeri* |
| DTO 019-I7 | *Aspergillus fischeri* | 2.519 | 2.670 | *Aspergillus fischeri* | 2.733 | 2.714 | *Aspergillus fischeri* | 2.477 | 2.663 | *Aspergillus fischeri* | 0 | 2.352 | *Aspergillus lentulus* |
| DTO 027-F8 | *Aspergillus fischeri* | 2.062 | 0 | *Aspergillus fischeri* | 0 | 2.662 | *Aspergillus fischeri* | 2.269 | 2.516 | *Aspergillus fischeri* | 0 | 0 | No peaks found |
| DTO 164-H5 | *Aspergillus fischeri* | 2.271 | 2.233 | *Aspergillus fischeri* | 2.532 | 2.592 | *Aspergillus fischeri* | 2.602 | 2.626 | *Aspergillus fischeri* | 2.354 | 0 | *Aspergillus lentulus* |
| DTO 204-F4 | *Aspergillus fumigatiaffinis* | 2.006 | 0 | *Aspergillus spinosus* | 2.206 | 2.225 | *Aspergillus spinosus* | 1.783 | 0 | *Aspergillus spinosus* | 2.067 | 2.026 | *Aspergillus spinosus* |
| DTO 010-D2 | *Aspergillus fumigatus* | 0 | 0 | No peaks found | 2.642 | 2.679 | *Aspergillus fumigatus* | 0 | 0 | No peaks found | 0 | 0 | No peaks found |
| DTO 012-C5 | *Aspergillus fumigatus* | 0 | 0 | No peaks found | 0 | 2.584 | *Aspergillus fumigatus* | 0 | 0 | No peaks found | 0 | 0 | No peaks found |
| DTO 028-D7 | *Aspergillus fumigatus* | 0 | 0 | No peaks found | 2.719 | 2.670 | *Aspergillus fumigatus* | 2.608 | 2.635 | *Aspergillus fumigatus* | 2.507 | 2.543 | *Aspergillus fumigatus* |
| DTO 041-C8 | *Aspergillus fumigatus* | 0 | 0 | No peaks found | 2.603 | 2.647 | *Aspergillus fumigatus* | 2.521 | 2.515 | *Aspergillus fumigatus* | 2.424 | 2.534 | *Aspergillus fumigatus* |
| DTO 064-C3 | *Aspergillus fumigatus* | 0 | 0 | No peaks found | 1.863 | 1.485 | *Aspergillus fumigatus* | 2.370 | 2.431 | *Aspergillus fumigatus* | 1.908 | 1.831 | *Aspergillus fumigatus* |
| DTO 073-I1 | *Aspergillus fumigatus* | 0 | 0 | No peaks found | 2.364 | 2.367 | *Aspergillus fumigatus* | 0 | 0 | No peaks found | 0 | 0 | No peaks found |
| DTO 075-I1 | *Aspergillus fumigatus* | 0 | 0 | No peaks found | 2.588 | 2.563 | *Aspergillus fumigatus* | 2.509 | 2.585 | *Aspergillus fumigatus* | 2.524 | 0 | *Aspergillus fumigatus* |
| DTO 076-A1 | *Aspergillus fumigatus* | 0 | 0 | No peaks found | 2.704 | 2.728 | *Aspergillus fumigatus* | 2.607 | 2.570 | *Aspergillus fumigatus* | 0 | 0 | No peaks found |
| DTO 082-E4 | *Aspergillus fumigatus* | 0 | 0 | No peaks found | 0 | 0 | No peaks found | 0 | 2.569 | *Aspergillus fumigatus* | 2.104 | 2.218 | *Aspergillus fumigatus* |
| DTO 084-D5 | *Aspergillus fumigatus* | 2.541 | 0 | *Aspergillus fumigatus* | 2.519 | 2.602 | *Aspergillus fumigatus* | 2.576 | 2.632 | *Aspergillus fumigatus* | 2.574 | 2.505 | *Aspergillus fumigatus* |
| DTO 085-F8 | *Aspergillus fumigatus* | 0 | 0 | No peaks found | 2.568 | 2.699 | *Aspergillus fumigatus* | 2.548 | 1.960 | *Aspergillus fumigatus* | 2.599 | 2.579 | *Aspergillus fumigatus* |
| DTO 086-C1 | *Aspergillus fumigatus* | 0 | 2.493 | *Aspergillus fumigatus* | 2.491 | 2.442 | *Aspergillus fumigatus* | 2.548 | 2.553 | *Aspergillus fumigatus* | 2.148 | 2.498 | *Aspergillus fumigatus* |
| DTO 086-C4 | *Aspergillus fumigatus* | 0 | 2.392 | *Aspergillus fumigatus* | 2.614 | 2.592 | *Aspergillus fumigatus* | 2.701 | 2.455 | *Aspergillus fumigatus* | 0 | 2.483 | *Aspergillus fumigatus* |
| DTO 091-I5 | *Aspergillus fumigatus* | 2.483 | 0 | *Aspergillus fumigatus* | 2.651 | 2.683 | *Aspergillus fumigatus* | 2.504 | 2.388 | *Aspergillus fumigatus* | 1.871 | 0 | *Aspergillus fumigatus* |
| DTO 094-E3 | *Aspergillus fumigatus* | 0 | 0 | No peaks found | 2.706 | 2.669 | *Aspergillus fumigatus* | 2.487 | 0 | *Aspergillus fumigatus* | 2.472 | 1.713 | *Aspergillus fumigatus* |
| DTO 094-E4 | *Aspergillus fumigatus* | 0 | 0 | No peaks found | 2.690 | 2.699 | *Aspergillus fumigatus* | 2.561 | 2.559 | *Aspergillus fumigatus* | 0 | 2.117 | *Aspergillus fumigatus* |
| DTO 109-A9 | *Aspergillus fumigatus* | 0 | 0 | No peaks found | 2.612 | 2.604 | *Aspergillus fumigatus* | 2.542 | 2.591 | *Aspergillus fumigatus* | 0 | 0 | No peaks found |
| DTO 124-E2 | *Aspergillus fumigatus* | 2.451 | 0 | *Aspergillus fumigatus* | 2.564 | 2.626 | *Aspergillus fumigatus* | 2.567 | 2.516 | *Aspergillus fumigatus* | 2.456 | 2.142 | *Aspergillus fumigatus* |
| DTO 150-E9 | *Aspergillus fumigatus* | 0 | 0 | No peaks found | 2.679 | 2.633 | *Aspergillus fumigatus* | 2.463 | 2.453 | *Aspergillus fumigatus* | 0 | 0 | No peaks found |
| DTO 152-G6 | *Aspergillus fumigatus* | 0 | 0 | No peaks found | 2.664 | 2.709 | *Aspergillus fumigatus* | 2.480 | 2.392 | *Aspergillus fumigatus* | 2.426 | 2.236 | *Aspergillus fumigatus* |
| DTO 167-H1 | *Aspergillus fumigatus* | 0 | 0 | No peaks found | 2.681 | 2.630 | *Aspergillus fumigatus* | 2.589 | 2.649 | *Aspergillus fumigatus* | 2.640 | 0 | *Aspergillus fumigatus* |
| DTO 244-G3 | *Aspergillus fumigatus* | 2.398 | 2.395 | *Aspergillus fumigatus* | 2.515 | 2.571 | *Aspergillus fumigatus* | 2.623 | 2.638 | *Aspergillus fumigatus* | 2.474 | 2.515 | *Aspergillus fumigatus* |
| DTO 271-A3 | *Aspergillus fumigatus* | 2.266 | 0 | *Aspergillus fumigatus* | 2.519 | 2.544 | *Aspergillus fumigatus* | 2.622 | 2.475 | *Aspergillus fumigatus* | 1.984 | 2.388 | *Aspergillus fumigatus* |
| DTO 171-A9 | *Aspergillus fumisynnematus* | 0 | 0 | No peaks found | 2.559 | 2.512 | *Aspergillus fumisynnematus* | 2.547 | 2.533 | *Aspergillus fumisynnematus* | 2.645 | 0 | *Aspergillus fumisynnematus* |
| DTO 171-B1 | *Aspergillus fumisynnematus* | 2.464 | 2.463 | *Aspergillus fumisynnematus* | 2.539 | 2.577 | *Aspergillus fumisynnematus* | 2.580 | 2.558 | *Aspergillus fumisynnematus* | 2.290 | 0 | *Aspergillus fumisynnematus* |
| DTO 227-I7 | *Aspergillus hiratsukae* | 0 | 2.467 | *Aspergillus hiratsukae* | 2.713 | 2.759 | *Aspergillus hiratsukae* | 2.533 | 2.748 | *Aspergillus hiratsukae* | 0 | 2.289 | *Aspergillus hiratsukae* |
| DTO 018-G7 | *Aspergillus lentulus* | 0 | 0 | No peaks found | 2.625 | 2.634 | *Aspergillus lentulus* | 0 | 0 | No peaks found | 2.585 | 0 | *Asp. fumisynnematus* |
| DTO 158-H4 | *Aspergillus lentulus* | 0 | 0 | No peaks found | 2.722 | 2.693 | *Aspergillus lentulus* | 2.650 | 2.539 | *Aspergillus lentulus* | 0 | 0 | No peaks found |
| DTO 179-A2 | *Aspergillus lentulus* | 0 | 0 | No peaks found | 2.569 | 2.565 | *Aspergillus lentulus* | 2.602 | 2.589 | *Aspergillus lentulus* | 0 | 0 | No peaks found |
| DTO 241-E4 | *Aspergillus niger* | 0 | 0 | No peaks found | 0 | 1.162 | No reliable ID | 0 | 0 | No peaks found | 1.129 | 1.215 | No reliable ID |
| DTO 003-H2 | *Aspergillus novofumigatus* | 0 | 0 | No peaks found | 0 | 2.296 | *Aspergillus lentulus* | 2.349 | 2.340 | *Aspergillus lentulus* | 2.227 | 0 | *Aspergillus* sp. 2 |
| DTO 258-D1 | *Aspergillus parasiticus* | 1.270 | 1.258 | No reliable ID | 1.602 | 1.506 | No reliable ID | 1.432 | 1.471 | No reliable ID | 1.164 | 1.149 | No reliable ID |
| DTO 001-E4 | *Aspergillus* sp. 1 | 0 | 0 | No peaks found | 0 | 2,214 | *Aspergillus fumisynnematus* | 0 | 2.395 | *Aspergillus lentulus* | 0 | 2.203 | *Aspergillus lentulus* |
| DTO 270-H3 | *Aspergillus terreus* | 0 | 0 | No peaks found | 1.561 | 1.563 | Not reliable ID | 1.486 | 1.479 | No reliable ID | 1.517 | 1.442 | No reliable ID |
| DTO 003-E7 | *Aspergillus thermomutatus* | 0 | 0 | No peaks found | 1.150 | 1.188 | No reliable ID | 1.022 | 1.188 | No reliable ID | 1.081 | 0.914 | No reliable ID |
| DTO 019-D8 | *Aspergillus udagawae* | 0 | 0 | No peaks found | 2.539 | 0 | *Aspergillus udagawae* | 0 | 2.341 | *Aspergillus udagawae* | 2.563 | 2.566 | *Aspergillus udagawae* |
| DTO 283-D3 | *Aspergillus udagawae* | 1.660 | 1.756 | *Aspergillus coreanus* | 2.536 | 2.568 | *Aspergillus udagawae* | 2.539 | 2.481 | *Aspergillus udagawae* | 2.182 | 2.321 | *Aspergillus udagawae* |
| DTO 084-G3 | *Penicillium glabrum* | 1.094 | 0 | No reliable ID | 1.311 | 1.147 | No reliable ID | 1.245 | 1.160 | No reliable ID | 1.412 | 1.375 | No reliable ID |
| DTO 149-A3 | *Penicillium rubens* | 0 | 0 | No peaks found | 1.092 | 1.054 | No reliable ID | 1.081 | 1.133 | No reliable ID | 1.288 | 1.019 | No reliable ID |
| DTO 442-G2 | *Talaromyces pinophilus* | 0 | 0 | No peaks found | 0.782 | 0.808 | No reliable ID | 0.819 | 0 | No reliable ID | 0 | 0.720 | No reliable ID |

1PC: growth on top of a polycarbonate filter, 2PC: growth between two polycarbonate filters, LC: liquid cultivation.

**Suppl. Table V: Identification results of the 50 test strains (set 3) against the BDAL database.**

| Strain no. | Molecular ID | SDA (control) | | | 1PC | | | 2PC | | | LC | | |
| --- | --- | --- | --- | --- | --- | --- | --- | --- | --- | --- | --- | --- | --- |
|  |  | Spot#1 | Spot#2 | MALDI ID | Spot#1 | Spot#2 | MALDI ID | Spot#1 | Spot#2 | MALDI ID | Spot#1 | Spot#2 | MALDI ID |
| DTO 057-D8 | *Aspergillus calidoustus* | 0 | 0 | No peaks found | 2.139 | 2.128 | *Aspergill ustus* | 0 | 0 | No peaks found | 1.779 | 1.762 | *Aspergill ustus* |
| DTO 131-G3 | *Aspergillus felis* | 0 | 1.501 | No reliable ID | 1.804 | 1.687 | *Aspergillus fumigatus* | 1.676 | 1.912 | *Aspergillus fumigatus* | 1.634 | 1.808 | *Aspergillus fumigatus* |
| DTO 176-F1 | *Aspergillus felis* | 1.609 | 0 | No reliable ID | 1.797 | 1.738 | *Aspergillus fumigatus* | 1.665 | 1.688 | No reliable ID | 1.573 | 1.758 | *Aspergillus fumigatus* |
| DTO 131-E3 | *Aspergillus felis* (contaminated with *A. fumigatus*) | 2.045 | 1.700 | *Aspergillus fumigatus* | 2.263 | 2.254 | *Aspergillus fumigatus* | 2.050 | 2.002 | *Aspergillus fumigatus* | 2.183 | 2.128 | *Aspergillus fumigatus* |
| DTO 010-G1 | *Aspergillus fischeri* | 1.059 | 0.980 | No reliable ID | 1.609 | 1.541 | No reliable ID | 1.633 | 1.685 | No reliable ID | 0 | 1.632 | No reliable ID |
| DTO 017-D1 | *Aspergillus fischeri* | 1.015 | 1.163 | No reliable ID | 1.414 | 1.487 | No reliable ID | 1.561 | 1.413 | No reliable ID | 1.648 | 1.840 | *Aspergillus fumigatus* |
| DTO 019-I7 | *Aspergillus fischeri* | 0.884 | 1.124 | No reliable ID | 1.731 | 1.730 | *Aspergillus fumigatus* | 1.062 | 1.455 | No reliable ID | 0 | 1.951 | *Aspergillus fumigatus* |
| DTO 027-F8 | *Aspergillus fischeri* | 0.960 | 0 | No reliable ID | 1.655 | 1.289 | No reliable ID | 1.432 | 0 | No reliable ID | 0 | 0 | No peaks found |
| DTO 164-H5 | *Aspergillus fischeri* | 1.210 | 1.031 | No reliable ID | 1.680 | 1.678 | No reliable ID | 1.611 | 1.637 | No reliable ID | 1.843 | 0 | *Aspergillus fumigatus* |
| DTO 204-F4 | *Aspergillus fumigatiaffinis* | 1.394 | 0 | No reliable ID | 1.669 | 1.757 | *Aspergillus fumigatus* | 1.425 | 0 | No reliable ID | 1.777 | 1.620 | *Aspergillus fumigatus* |
| DTO 010-D2 | *Aspergillus fumigatus* | 0 | 0 | No peaks found | 2.239 | 2.395 | *Aspergillus fumigatus* | 0 | 0 | No peaks found | 0 | 0 | No peaks found |
| DTO 012-C5 | *Aspergillus fumigatus* | 0 | 0 | No peaks found | 0 | 2.051 | *Aspergillus fumigatus* | 0 | 0 | No peaks found | 0 | 0 | No peaks found |
| DTO 028-D7 | *Aspergillus fumigatus* | 0 | 0 | No peaks found | 2.340 | 2.390 | *Aspergillus fumigatus* | 2.276 | 2.269 | *Aspergillus fumigatus* | 2.242 | 2.291 | *Aspergillus fumigatus* |
| DTO 041-C8 | *Aspergillus fumigatus* | 0 | 0 | No peaks found | 2.438 | 2.405 | *Aspergillus fumigatus* | 2.147 | 2.091 | *Aspergillus fumigatus* | 2.202 | 2.383 | *Aspergillus fumigatus* |
| DTO 064-C3 | *Aspergillus fumigatus* | 0 | 0 | No peaks found | 1.177 | 1.214 | No reliable ID | 1.808 | 1.989 | *Aspergillus fumigatus* | 1.496 | 1.672 | No reliable ID |
| DTO 073-I1 | *Aspergillus fumigatus* | 0 | 0 | No peaks found | 1.827 | 2.007 | *Aspergillus fumigatus* | 0 | 0 | No peaks found | 0 | 0 | No peaks found |
| DTO 075-I1 | *Aspergillus fumigatus* | 0 | 0 | No peaks found | 2.216 | 2.284 | *Aspergillus fumigatus* | 2.148 | 2.289 | *Aspergillus fumigatus* | 2.251 | 0 | *Aspergillus fumigatus* |
| DTO 076-A1 | *Aspergillus fumigatus* | 0 | 0 | No peaks found | 2.357 | 2.445 | *Aspergillus fumigatus* | 2.289 | 2.268 | *Aspergillus fumigatus* | 0 | 0 | No peaks found |
| DTO 082-E4 | *Aspergillus fumigatus* | 0 | 0 | No peaks found | 0 | 0 | No peaks found | 0 | 2.204 | *Aspergillus fumigatus* | 1.828 | 1.900 | *Aspergillus fumigatus* |
| DTO 084-D5 | *Aspergillus fumigatus* | 2.202 | 0 | *Aspergillus fumigatus* | 2.072 | 2.164 | *Aspergillus fumigatus* | 2.194 | 2.333 | *Aspergillus fumigatus* | 1.900 | 2.150 | *Aspergillus fumigatus* |
| DTO 085-F8 | *Aspergillus fumigatus* | 0 | 0 | No peaks found | 2.180 | 2.273 | *Aspergillus fumigatus* | 2.127 | 1.755 | *Aspergillus fumigatus* | 2.386 | 2.336 | *Aspergillus fumigatus* |
| DTO 086-C1 | *Aspergillus fumigatus* | 0 | 2.027 | *Aspergillus fumigatus* | 2.123 | 1.982 | *Aspergillus fumigatus* | 2.163 | 2.022 | *Aspergillus fumigatus* | 2.040 | 2.405 | *Aspergillus fumigatus* |
| DTO 086-C4 | *Aspergillus fumigatus* | 0 | 1.998 | *Aspergillus fumigatus* | 2.303 | 2.254 | *Aspergillus fumigatus* | 2.379 | 2.170 | *Aspergillus fumigatus* | 0 | 2.270 | *Aspergillus fumigatus* |
| DTO 091-I5 | *Aspergillus fumigatus* | 2.076 | 0 | *Aspergillus fumigatus* | 2.373 | 2.348 | *Aspergillus fumigatus* | 1.997 | 1.988 | *Aspergillus fumigatus* | 1.912 | 0 | *Aspergillus fumigatus* |
| DTO 094-E3 | *Aspergillus fumigatus* | 0 | 0 | No peaks found | 2.321 | 2.351 | *Aspergillus fumigatus* | 1.980 | 0 | *Aspergillus fumigatus* | 2.123 | 1.115 | *Aspergillus fumigatus* |
| DTO 094-E4 | *Aspergillus fumigatus* | 0 | 0 | No peaks found | 2.371 | 2.421 | *Aspergillus fumigatus* | 2.169 | 2.020 | *Aspergillus fumigatus* | 0 | 2.080 | *Aspergillus fumigatus* |
| DTO 109-A9 | *Aspergillus fumigatus* | 0 | 0 | No peaks found | 2.176 | 2.178 | *Aspergillus fumigatus* | 2.020 | 2.164 | *Aspergillus fumigatus* | 0 | 0 | No peaks found |
| DTO 124-E2 | *Aspergillus fumigatus* | 1.791 | 0 | *Aspergillus fumigatus* | 2.168 | 2.283 | *Aspergillus fumigatus* | 2.063 | 2.071 | *Aspergillus fumigatus* | 2.183 | 1.743 | *Aspergillus fumigatus* |
| DTO 150-E9 | *Aspergillus fumigatus* | 0 | 0 | No peaks found | 2.337 | 2.325 | *Aspergillus fumigatus* | 2.095 | 2.093 | *Aspergillus fumigatus* | 0 | 0 | No peaks found |
| DTO 152-G6 | *Aspergillus fumigatus* | 0 | 0 | No peaks found | 2.383 | 2.354 | *Aspergillus fumigatus* | 2.032 | 1.915 | *Aspergillus fumigatus* | 2.240 | 2.066 | *Aspergillus fumigatus* |
| DTO 167-H1 | *Aspergillus fumigatus* | 0 | 0 | No peaks found | 2.417 | 2.210 | *Aspergillus fumigatus* | 2.334 | 2.373 | *Aspergillus fumigatus* | 2.422 | 0 | *Aspergillus fumigatus* |
| DTO 244-G3 | *Aspergillus fumigatus* | 1.884 | 1.956 | *Aspergillus fumigatus* | 2.064 | 2.081 | *Aspergillus fumigatus* | 2.272 | 2.352 | *Aspergillus fumigatus* | 2.297 | 2.337 | *Aspergillus fumigatus* |
| DTO 271-A3 | *Aspergillus fumigatus* | 1.931 | 0 | *Aspergillus fumigatus* | 2.296 | 2.138 | *Aspergillus fumigatus* | 2.326 | 2.248 | *Aspergillus fumigatus* | 1.724 | 2.235 | *Aspergillus fumigatus* |
| DTO 171-A9 | *Aspergillus fumisynnematus* | 0 | 0 | No peaks found | 1.768 | 1.624 | *Aspergillus fumigatus* | 1.603 | 1.545 | No reliable ID | 1.712 | 0 | *Aspergillus fumigatus* |
| DTO 171-B1 | *Aspergillus fumisynnematus* | 1.401 | 1.437 | No reliable ID | 1.343 | 1.451 | No reliable ID | 1.622 | 1.621 | No reliable ID | 1.687 | 0 | No reliable ID |
| DTO 227-I7 | *Aspergillus hiratsukae* | 1.278 | 0 | No reliable ID | 1.472 | 1.494 | No reliable ID | 1.455 | 1.571 | No reliable ID | 0 | 1.318 | No reliable ID |
| DTO 018-G7 | *Aspergillus lentulus* | 0 | 0 | No peaks found | 1.810 | 1.885 | *Aspergillus fumigatus* | 0 | 0 | No peaks found | 1.750 | 0 | *Aspergillus fumigatus* |
| DTO 158-H4 | *Aspergillus lentulus* | 0 | 0 | No peaks found | 1.821 | 1.732 | *Aspergillus fumigatus* | 1.500 | 1.466 | No reliable ID | 0 | 0 | No peaks found |
| DTO 179-A2 | *Aspergillus lentulus* | 0 | 0 | No peaks found | 1.809 | 1.737 | *Aspergillus fumigatus* | 1.548 | 1.722 | *Aspergillus fumigatus* | 0 | 0 | No peaks found |
| DTO 241-E4 | *Aspergillus niger* | 0 | 0 | No peaks found | 0 | 1.776 | *Aspergillus niger* | 0 | 0 | No peaks found | 2.257 | 2.277 | *Aspergillus niger* |
| DTO 003-H2 | *Aspergillus novofumigatus* | 0 | 0 | No peaks found | 0 | 1.457 | No reliable ID | 1.415 | 1.441 | No reliable ID | 1.556 | 0 | No reliable ID |
| DTO 258-D1 | *Aspergillus parasiticus* | 1.511 | 1.790 | *Aspergillus parasiticus* | 2.012 | 2.190 | *Aspergillus parasiticus* | 1.959 | 1.905 | *Aspergillus parasiticus* | 1.477 | 1.206 | No reliable ID |
| DTO 001-E4 | *Aspergillus* sp. 1 | 0 | 0 | No peaks found | 0 | 1.686 | No reliable ID | 0 | 1.639 | No reliable ID | 0 | 1.698 | No reliable ID |
| DTO 270-H3 | *Aspergillus terreus* | 0 | 0 | No peaks found | 2.183 | 2.166 | *Aspergillus terreus* | 1.985 | 2.230 | *Aspergillus terreus* | 2.432 | 2.401 | *Aspergillus terreus* |
| DTO 003-E7 | *Aspergillus thermomutatus* | 0 | 0 | No peaks found | 1.354 | 1.336 | No reliable ID | 1.425 | 1.267 | No reliable ID | 1.510 | 1.448 | No reliable ID |
| DTO 019-D8 | *Aspergillus udagawae* | 0 | 0 | No peaks found | 1.676 | 0 | No reliable ID | 0 | 1.290 | No reliable ID | 1.671 | 1.762 | *Aspergillus fumigatus* |
| DTO 283-D3 | *Aspergillus udagawae* | 1.049 | 0.960 | No reliable ID | 1.495 | 1.558 | No reliable ID | 1.564 | 1.666 | No reliable ID | 1.174 | 1.240 | No reliable ID |
| DTO 084-G3 | *Penicillium glabrum* | 1.729 | 0 | *Penicillium glabrum* | 1.589 | 1.776 | *Penicillium glabrum* | 1.786 | 1.519 | *Penicillium glabrum* | 2.103 | 2.136 | *Penicillium glabrum* |
| DTO 149-A3 | *Penicillium rubens* | 0 | 0 | No peaks found | 2.064 | 1.940 | *Penicillium chrysogenum* | 1.714 | 1.597 | *Penicillium chrysogenum* | 2.163 | 2.142 | *Penicillium chrysogenum* |
| DTO 442-G2 | *Talaromyces pinophilus* | 0 | 0 | No peaks found | 1.728 | 1.611 | *Talaromyces funiculosus* | 0 | 1.817 | *Tal. funiculosus* | 1.909 | 0 | *Talaromyces funiculosus* |

1PC: growth on top of a polycarbonate filter, 2PC: growth between two polycarbonate filters, LC: liquid cultivation.

**Suppl. Table VI: Identification results of the 50 test strains (set 3) against the combined in-house *Aspergillus* section *Fumigati* database and BDAL.**

| Strain no. | Molecular ID | SDA (control) | | | 1PC | | | 2PC | | | LC | | |
| --- | --- | --- | --- | --- | --- | --- | --- | --- | --- | --- | --- | --- | --- |
|  |  | Spot #1 | Spot #2 | MALDI ID | Spot #1 | Spot #2 | MALDI ID | Spot #1 | Spot#2 | MALDI ID | Spot#1 | Spot#2 | MALDI ID |
| DTO 057-D8 | *Aspergillus calidoustus* | 0 | 0 | No peaks found | 2.080 | 2.066 | *Aspergillus ustus* | 0 | 0 | No peaks found | 1.681 | 1.741 | *Aspergillus ustus* |
| DTO 131-G3 | *Aspergillus felis* | 1.890 | 0 | *Aspergillus felis* | 2.257 | 1.942 | *Aspergillus felis* | 2.463 | 2.403 | *Aspergillus felis* | 2.283 | 1.982 | *Aspergillus felis* |
| DTO 176-F1 | *Aspergillus felis* | 2.184 | 0 | *Aspergillus felis* | 2.324 | 1.909 | *Aspergillus felis / A. fischeri* | 2.343 | 1.854 | *Aspergillus felis / A. udagawae* | 1.688 | 2.131 | *Aspergillus felis* |
| DTO 131-E3 | *Aspergillus felis* (contaminated with *A. fumigatus*) | 1.933 | 1.895 | *Aspergillus fumigatus* | 1.842 | 2.473 | *Aspergillus fumigatus* | 2.287 | 2.276 | *Aspergillus fumigatus* | 2.088 | 2.234 | *Aspergillus fumigatus* |
| DTO 010-G1 | *Aspergillus fischeri* | 2.316 | 2.293 | *Aspergillus fischeri* | 2.491 | 2.433 | *Aspergillus fischeri* | 2.427 | 2.496 | *Aspergillus fischeri* | 0 | 2.236 | *Aspergillus fischeri* |
| DTO 017-D1 | *Aspergillus fischeri* | 2.194 | 2.284 | *Aspergillus fischeri* | 2.470 | 2.446 | *Aspergillus fischeri* | 2.337 | 2.490 | *Aspergillus fischeri* | 2.179 | 2.285 | *Aspergillus fischeri* |
| DTO 019-I7 | *Aspergillus fischeri* | 2.508 | 2.370 | *Aspergillus fischeri* | 2.506 | 2.542 | *Aspergillus fischeri* | 2.350 | 2.178 | *Aspergillus fischeri* | 0 | 2.329 | *Aspergillus fischeri* |
| DTO 027-F8 | *Aspergillus fischeri* | 1.738 | 0 | *Aspergillus fischeri* | 0 | 2.367 | *Aspergillus fischeri* | 1.806 | 2.161 | *Aspergillus fischeri* | 0 | 0 | No peaks found |
| DTO 164-H5 | *Aspergillus fischeri* | 1.966 | 2.106 | *Aspergillus fischeri* | 2.452 | 2.503 | *Aspergillus fischeri* | 2.386 | 2.455 | *Aspergillus fischeri* | 1.984 | 0 | *Aspergillus lacinosus* |
| DTO 204-F4 | *Aspergillus* *fumigatiaffinis* | 1.802 | 0 | *Aspergillus udagawae* | 2.074 | 2.044 | *Aspergillus coreanus / A. spinosus* | 1.657 | 0 | No reliable ID | 1.884 | 1.887 | *Aspergillus spinosus / A. viridinutans* |
| DTO 010-D2 | *Aspergillus fumigatus* | 0 | 0 | No peaks found | 2.172 | 2.454 | *Aspergillus fumigatus* | 0 | 0 | No peaks found | 0 | 0 | No peaks found |
| DTO 012-C5 | *Aspergillus fumigatus* | 0 | 0 | No peaks found | 0 | 2.345 | *Aspergillus fumigatus* | 0 | 0 | No peaks found | 0 | 0 | No peaks found |
| DTO 028-D7 | *Aspergillus fumigatus* | 0 | 0 | No peaks found | 2.324 | 2.535 | *Aspergillus fumigatus* | 2.452 | 2.338 | *Aspergillus fumigatus* | 2.425 | 2.363 | *Aspergillus fumigatus* |
| DTO 041-C8 | *Aspergillus fumigatus* | 0 | 0 | No peaks found | 2.501 | 2.443 | *Aspergillus fumigatus* | 2.242 | 2.223 | *Aspergillus fumigatus* | 2.127 | 2.302 | *Aspergillus fumigatus* |
| DTO 064-C3 | *Aspergillus fumigatus* | 0 | 0 | No peaks found | 1.543 | 1.653 | No reliable ID | 1.895 | 2.229 | *Aspergillus fumigatus* | 1.736 | 1.679 | *Aspergillus fumigatus* |
| DTO 073-I1 | *Aspergillus fumigatus* | 0 | 0 | No peaks found | 2.295 | 2.135 | *Aspergillus fumigatus* | 0 | 0 | No peaks found | 0 | 0 | No peaks found |
| DTO 075-I1 | *Aspergillus fumigatus* | 0 | 0 | No peaks found | 2.340 | 2.446 | *Aspergillus fumigatus* | 2.222 | 2.336 | *Aspergillus fumigatus* | 2.356 | 0 | *Aspergillus fumigatus* |
| DTO 076-A1 | *Aspergillus fumigatus* | 0 | 0 | No peaks found | 2.508 | 2.552 | *Aspergillus fumigatus* | 2.381 | 2.427 | *Aspergillus fumigatus* | 0 | 0 | No peaks found |
| DTO 082-E4 | *Aspergillus fumigatus* | 0 | 0 | No peaks found | 0 | 0 | No peaks found | 0 | 2.333 | *Aspergillus fumigatus* | 2.026 | 2.004 | *Aspergillus fumigatus* |
| DTO 084-D5 | *Aspergillus fumigatus* | 2.398 | 0 | *Aspergillus fumigatus* | 1.952 | 2.417 | *Aspergillus fumigatus* | 2.363 | 2.400 | *Aspergillus fumigatus* | 2.162 | 2.216 | *Aspergillus fumigatus* |
| DTO 085-F8 | *Aspergillus fumigatus* | 0 | 0 | No peaks found | 2.392 | 2.360 | *Aspergillus fumigatus* | 1.534 | 2.315 | *Aspergillus fumigatus* | 2.518 | 2.325 | *Aspergillus fumigatus* |
| DTO 086-C1 | *Aspergillus fumigatus* | 0 | 2.163 | *Aspergillus fumigatus* | 2.323 | 2.290 | *Aspergillus fumigatus* | 2.287 | 2.240 | *Aspergillus fumigatus* | 2.041 | 2.329 | *Aspergillus fumigatus* |
| DTO 086-C4 | *Aspergillus fumigatus* | 0 | 2.215 | *Aspergillus fumigatus* | 2.429 | 2.073 | *Aspergillus fumigatus* | 2.351 | 2.329 | *Aspergillus fumigatus* | 0 | 2.214 | *Aspergillus fumigatus* |
| DTO 091-I5 | *Aspergillus fumigatus* | 2.338 | 0 | *Aspergillus fumigatus* | 2.458 | 2.466 | *Aspergillus fumigatus* | 2.302 | 2.074 | *Aspergillus fumigatus* | 1.766 | 0 | *Aspergillus fumigatus* |
| DTO 094-E3 | *Aspergillus fumigatus* | 0 | 0 | No peaks found | 2.411 | 2.496 | *Aspergillus fumigatus* | 2.286 | 0 | *Aspergillus fumigatus* | 1.559 | 2.231 | *Aspergillus fumigatus* |
| DTO 094-E4 | *Aspergillus fumigatus* | 0 | 0 | No peaks found | 2.503 | 2.504 | *Aspergillus fumigatus* | 2.300 | 2.327 | *Aspergillus fumigatus* | 0 | 1.960 | *Aspergillus fumigatus* |
| DTO 109-A9 | *Aspergillus fumigatus* | 0 | 0 | No peaks found | 1.705 | 2.327 | *Aspergillus fumigatus* | 2.275 | 2.368 | *Aspergillus fumigatus* | 0 | 0 | No peaks found |
| DTO 124-E2 | *Aspergillus fumigatus* | 2.056 | 0 | *Aspergillus fumigatus* | 2.523 | 2.375 | *Aspergillus fumigatus* | 2.090 | 1.927 | *Aspergillus fumigatus* | 2.155 | 1.983 | *Aspergillus fumigatus* |
| DTO 150-E9 | *Aspergillus fumigatus* | 0 | 0 | No peaks found | 2.500 | 2.520 | *Aspergillus fumigatus* | 2.115 | 2.329 | *Aspergillus fumigatus* | 0 | 0 | No peaks found |
| DTO 152-G6 | *Aspergillus fumigatus* | 0 | 0 | No peaks found | 2.415 | 1.856 | *Aspergillus fumigatus* | 1.593 | 2.093 | *Aspergillus fumigatus* | 2.213 | 2.125 | *Aspergillus fumigatus* |
| DTO 167-H1 | *Aspergillus fumigatus* | 0 | 0 | No peaks found | 2.576 | 2.410 | *Aspergillus fumigatus* | 2.369 | 2.393 | *Aspergillus fumigatus* | 2.358 | 0 | *Aspergillus fumigatus* |
| DTO 244-G3 | *Aspergillus fumigatus* | 2.276 | 2.193 | *Aspergillus fumigatus* | 2.244 | 2.222 | *Aspergillus fumigatus* | 2.429 | 2.328 | *Aspergillus fumigatus* | 2.210 | 2.387 | *Aspergillus fumigatus* |
| DTO 271-A3 | *Aspergillus fumigatus* | 0 | 2.000 | *Aspergillus fumigatus* | 2.218 | 2.331 | *Aspergillus fumigatus* | 2.320 | 2.487 | *Aspergillus fumigatus* | 1.811 | 2.110 | *Aspergillus fumigatus* |
| DTO 171-A9 | *Aspergillus fumisynnematus* | 0 | 0 | No peaks found | 2.387 | 2.266 | *Aspergillus fumisynnematus* | 2.350 | 2.450 | *Aspergillus fumisynnematus* | 2.433 | 0 | *Aspergillus fumisynnematus* |
| DTO 171-B1 | *Aspergillus fumisynnematus* | 2.309 | 2.382 | *Aspergillus fumisynnematus* | 1.893 | 2.213 | *Aspergillus fumisynnematus* | 2.267 | 1.962 | *Aspergillus fumisynnematus / A. fischeri* | 2.090 | 0 | *Aspergillus fumisynnematus* |
| DTO 227-I7 | *Aspergillus hiratsukae* | 0 | 2.307 | *Aspergillus hiratsukae* | 2.418 | 2.361 | *Aspergillus hiratsukae* | 2.305 | 1.815 | *Aspergillus hiratsukae* | 0 | 1.937 | *Aspergillus fennelliae* |
| DTO 018-G7 | *Aspergillus lentulus* | 0 | 0 | No peaks found | 2.437 | 2.430 | *Aspergillus lentulus* | 0 | 0 | No peaks found | 0 | 2.408 | *Aspergillus fumisynnematus* |
| DTO 158-H4 | *Aspergillus lentulus* | 0 | 0 | No peaks found | 2.638 | 2.516 | *Aspergillus lentulus* | 2.423 | 2.274 | *Aspergillus lentulus* | 0 | 0 | No peaks found |
| DTO 179-A2 | *Aspergillus lentulus* | 0 | 0 | No peaks found | 2.420 | 2.432 | *Aspergillus lentulus* | 2.407 | 2.386 | *Aspergillus lentulus* | 0 | 0 | No peaks found |
| DTO 241-E4 | *Aspergillus niger* | 0 | 0 | No peaks found | 0 | 1.861 | *Aspergillus niger* | 0 | 0 | No peaks found | 2.337 | 2.101 | *Aspergillus niger* |
| DTO 003-H2 | *Aspergillus novofumigatus* | 0 | 0 | No peaks found | 0 | 2.225 | *Aspergillus lentulus* | 2.071 | 2.073 | *Aspergillus lentulus* | 2.042 | 0 | *Aspergillus* sp. 2 |
| DTO 258-D1 | *Aspergillus parasiticus* | 1.660 | 1.671 | No reliable ID | 1.876 | 2.052 | *Aspergillus parasiticus* | 2.061 | 1.906 | *Aspergillus parasiticus* | 1.474 | 1.585 | No reliable ID |
| DTO 001-E4 | *Aspergillus* sp. 1 | 0 | 0 | No peaks found | 0 | 2.121 | *Aspergillus fumisynnematus* | 0 | 2.344 | *Aspergillus fumisynnematus* | 0 | 2.018 | *Aspergillus* sp. 2 |
| DTO 270-H3 | *Aspergillus terreus* | 0 | 0 | No peaks found | 1.886 | 2.096 | *Aspergillus terreus* | 2.231 | 2.228 | *Aspergillus terreus* | 2.409 | 2.460 | *Aspergillus terreus* |
| DTO 003-E7 | *Aspergillus thermomutatus* | 0 | 0 | No peaks found | 1.225 | 1.417 | No reliable ID | 1.418 | 1.334 | No reliable ID | 1.692 | 1.495 | No reliable ID |
| DTO 019-D8 | *Aspergillus udagawae* | 0 | 0 | No peaks found | 0 | 2.187 | *Aspergillus udagawae* | 2.132 | 0 | *Aspergillus udagawae* | 2.201 | 2.322 | *Aspergillus udagawae* |
| DTO 283-D3 | *Aspergillus udagawae* | 1.830 | 1.855 | *Aspergillus coreanus / A. udagawae* | 2.403 | 2.368 | *Aspergillus udagawae* | 2.274 | 2.143 | *Aspergillus udagawae* | 2.138 | 2.199 | *Aspergillus udagawae* |
| DTO 084-G3 | *Penicillium glabrum* | 1.564 | 0 | No reliable ID | 1.414 | 1.697 | No reliable ID | 1.371 | 1.502 | No reliable ID | 2.093 | 2.140 | *Penicillium glabrum* |
| DTO 149-A3 | *Penicillium rubens* | 0 | 0 | No peaks found | 1.824 | 1.774 | *Penicillium chrysogenum* | 1.781 | 1.934 | *Penicillium chrysogenum* | 2.051 | 2.045 | *Penicillium chrysogenum* |
| DTO 442-G2 | *Talaromyces pinophilus* | 0 | 0 | No peaks found | 1.753 | 1.815 | *Talaromyces funiculosus* | 0 | 1.998 | *Talaromyces funiculosus* | 0 | 1.703 | *Talaromyces funiculosus* |

1PC: growth on top of a polycarbonate filter, 2PC: growth between two polycarbonate filters, LC: liquid cultivation.

**Suppl. Table VII: Identification errors associated with initial validation (strain set 3).**

|  | **Cultivation method** | **Strain no.** | **Molecular identification** | **MALDI-TOF MS identification** | **Score** | **Error (minor/major)** |
| --- | --- | --- | --- | --- | --- | --- |
| **In-house database** | Control | DTO 283-D3 | *Aspergillus udagawae* | *Aspergillus coreanus* | 1.756 | Major |
|  |  | DTO 204-F4 | *Aspergillus fumigatiaffinis* | *Aspergillus spinosus* | 2.006 | Major |
|  | 1PC | DTO 204-F4 | *Aspergillus fumigatiaffinis* | *Aspergillus spinosus* | 2.225 | Major |
|  | 2PC | DTO 204-F4 | *Aspergillus fumigatiaffinis* | *Aspergillus spinosus* | 1.783 | Major |
|  | LC | DTO 019-I7 | *Aspergillus fischeri* | *Aspergillus lentulus* | 2.352 | Minor |
|  |  | DTO 018-G7 | *Aspergillus lentulus* | *Aspergillus fumisynnematus* | 2.585 | Minor |
|  |  | DTO 164-H5 | *Aspergillus fischeri* | *Aspergillus lentulus* | 2.354 | Minor |
|  |  | DTO 010-G1 | *Aspergillus fischeri* | *Aspergillus lentulus* | 2.350 | Minor |
|  |  | DTO 204-F4 | *Aspergillus fumigatiaffinis* | *Aspergillus spinosus* | 2.067 | Major |
| **All databases** | Control | DTO 204-F4 | *Aspergillus fumigatiaffinis* | *Aspergillus udagawae* | 1.802 | Major |
|  | 1PC | DTO 204-F4 | *Aspergillus fumigatiaffinis* | *Aspergillus coreanus* | 2.074 | Minor |
|  | LC | DTO 204-F4 | *Aspergillus fumigatiaffinis* | *Aspergillus viridinutans* | 1.887 | Major |
|  |  | DTO 164-H5 | *Aspergillus fischeri* | *Aspergillus lacinosus* | 1.984 | Minor |
|  |  | DTO 227-I7 | *Aspergillus hiratsukae* | *Aspergillus fennelliae* | 1.937 | Major |
|  |  | DTO 018-G7 | *Aspergillus lentulus* | *Aspergillus fumisynnematus* | 2.408 | Minor |

1PC: growth on a polycarbonate filter, 2PC: growth between two polycarbonate filters, LC: Liquid cultivation.

**Suppl. Table VIII: False positive identifications (species not included in database) of test strains (set 3).**

|  | **Cultivation method** | **Strain no.** | **Molecular identification** | **MALDI-TOF MS identification** | **Score** | **Error (minor/major)** |
| --- | --- | --- | --- | --- | --- | --- |
| **In-house database** | 1PC | DTO 001-E4 | *Aspergillus* sp. 1 | *Aspergillus fumisynnematus* | 2.214 | Minor |
|  |  | DTO 003-H2 | *Aspergillus novofumigatus* | *Aspergillus lentulus* | 2.296 | Minor |
|  | 2PC | DTO 001-E4 | *Aspergillus* sp. 1 | *Aspergillus lentulus* | 2.395 | Minor |
|  |  | DTO 003-H2 | *Aspergillus novofumigatus* | *Aspergillus lentulus* | 2.349 | Minor |
|  | LC | DTO 001-E4 | *Aspergillus* sp. 1 | *Aspergillus lentulus* | 2.203 | Minor |
|  |  | DTO 003-H2 | *Aspergillus novofumigatus* | *Aspergillus* sp. 2 | 2.227 | Minor |
| **Bruker database** | 1PC | DTO 018-G7 | *Aspergillus lentulus* | *Aspergillus fumigatus* | 1.885 | Major |
|  |  | DTO 158-H4 | *Aspergillus lentulus* | *Aspergillus fumigatus* | 1.821 | Major |
|  |  | DTO 179-A2 | *Aspergillus lentulus* | *Aspergillus fumigatus* | 1.809 | Major |
|  |  | DTO 019-I7 | *Aspergillus fischeri* | *Aspergillus fumigatus* | 1.731 | Major |
|  |  | DTO 171-A9 | *Aspergillus fumisynnematus* | *Aspergillus fumigatus* | 1.768 | Major |
|  |  | DTO 176-F1 | *Aspergillus felis* | *Aspergillus fumigatus* | 1.797 | Major |
|  |  | DTO 149-A3 | *Penicillium rubens* | *Penicillium chrysogenum* | 2.064 | Minor |
|  |  | DTO 057-D8 | *Aspergillus calidoustus* | *Aspergill ustus* | 2.139 | Major |
|  |  | DTO 204-F4 | *Aspergillus fumigatiaffinis* | *Aspergillus fumigatus* | 1.757 | Major |
|  |  | DTO 442-G2 | *Talaromyces pinophilus* | *Talaromyces funiculosus* | 1.728 | Major |
|  |  | DTO 131-G3 | *Aspergillus felis* | *Aspergillus fumigatus* | 1.804 | Major |
|  | 2PC | DTO 131-G3 | *Aspergillus felis* | *Aspergillus fumigatus* | 1.912 | Major |
|  |  | DTO 179-A2 | *Aspergillus lentulus* | *Aspergillus fumigatus* | 1.722 | Major |
|  |  | DTO 442-G2 | *Talaromyces pinophilus* | *Talaromyces funiculosus* | 1.817 | Major |
|  |  | DTO 149-A3 | *Penicillium rubens* | *Penicillium chrysogenum* | 1.714 | Minor |
|  | LC | DTO 017-D1 | *Aspergillus fischeri* | *Aspergillus fumigatus* | 1.840 | Major |
|  |  | DTO 018-G7 | *Aspergillus lentulus* | *Aspergillus fumigatus* | 1.750 | Major |
|  |  | DTO 019-D8 | *Aspergillus udagawae* | *Aspergillus fumigatus* | 1.762 | Major |
|  |  | DTO 019-I7 | *Aspergillus fischeri* | *Aspergillus fumigatus* | 1.951 | Major |
|  |  | DTO 164-H5 | *Aspergillus fischeri* | *Aspergillus fumigatus* | 1.843 | Major |
|  |  | DTO 171-A9 | *Aspergillus fumisynnematus* | *Aspergillus fumigatus* | 1.712 | Major |
|  |  | DTO 176-F1 | *Aspergillus felis* | *Aspergillus fumigatus* | 1.758 | Major |
|  |  | DTO 149-A3 | *Penicillium rubens* | *Penicillium chrysogenum* | 2.163 | Minor |
|  |  | DTO 057-D8 | *Aspergillus calidoustus* | *Aspergill ustus* | 1.779 | Major |
|  |  | DTO 204-F4 | *Aspergillus fumigatiaffinis* | *Aspergillus fumigatus* | 1.777 | Major |
|  |  | DTO 442-G2 | *Talaromyces pinophilus* | *Talaromyces funiculosus* | 1.909 | Major |
|  |  | DTO 131-G3 | *Aspergillus felis* | *Aspergillus fumigatus* | 1.808 | Major |
| **All database** | 1PC | DTO 001-E4 | *Aspergillus* sp. 1 | *Aspergillus fumisynnematus* | 2.121 | Minor |
|  |  | DTO 003-H2 | *Aspergillus novofumigatus* | *Aspergillus lentulus* | 2.225 | Minor |
|  |  | DTO 149-A3 | *Penicillium rubens* | *Penicillium chrysogenum* | 1.824 | Minor |
|  |  | DTO 442-G2 | *Talaromyces pinophilus* | *Talaromyces funiculosus* | 1.815 | Major |
|  |  | DTO 057-D8 | *Aspergillus calidoustus* | *Aspergillus ustus* | 2.080 | Major |
|  | 2PC | DTO 001-E4 | *Aspergillus* sp. 1 | *Aspergillus fumisynnematus* | 2.344 | Minor |
|  |  | DTO 003-H2 | *Aspergillus novofumigatus* | *Aspergillus lentulus* | 2.073 | Minor |
|  |  | DTO 442-G2 | *Talaromyces pinophilus* | *Talaromyces funiculosus* | 1.909 | Major |
|  |  | DTO 149-A3 | *Penicillium rubens* | *Penicillium chrysogenum* | 1.934 | Minor |
|  | LC | DTO 001-E4 | *Aspergillus* sp. 1 | *Aspergillus* sp. 2 | 2.018 | Minor |
|  |  | DTO 003-H2 | *Aspergillus novofumigatus* | *Aspergillus* sp. 2 | 2.042 | Minor |
|  |  | DTO 149-A3 | *Penicillium rubens* | *Penicillium chrysogenum* | 2.051 | Minor |
|  |  | DTO 057-D8 | *Aspergillus calidoustus* | *Aspergillus ustus* | 1.741 | Major |
|  |  | DTO 442-G2 | *Talaromyces pinophilus* | *Talaromyces funiculosus* | 1.909 | Major |

1PC: growth on a polycarbonate filter, 2PC: growth between two polycarbonate filters, LC: liquid cultivation.

**Suppl. Table IX: MALDI-TOF MS identifications of Indonesian clinical isolates.**

| DTO no. | Molecular ID | All databases #1 match | Score | All databases #2 match | Score | LC # 1 match | Score | LC #2 Match | Score2 | 1PC #1 match | Score | 1PC #2 match | Score | 2PC #1 match | Score | 2PC #2 match | Score |
| --- | --- | --- | --- | --- | --- | --- | --- | --- | --- | --- | --- | --- | --- | --- | --- | --- | --- |
| DTO 310-B1 | *A. flavus* | *A. flavus* | 1.985 | *A. minisclerotigenes* | 1.925 | *A. flavus* | 1.985 | *A. flavus* | 1.918 | *A. flavus* | 1.814 | *A. flavus* | 1.763 | *A. minisclerotigenes* | 1.925 | *A. flavus* | 1.893 |
|  |  | *A. flavus* | 2.447 | *A. flavus* | 2.417 | *A. flavus* | 2.316 | *A. flavus* | 2.212 | *A. flavus* | 2.377 | *A. flavus* | 2.328 | *A. flavus* | 2.447 | *A. flavus* | 2.417 |
| DTO 310-B3 | *A. fumigatus* | *A. fumigatus* | 2.072 | *A. nishimurae* | 1.834 | *A. fumigatus* | 2.072 | *A. fumigatus* | 1.831 | No reliable identification | 1.663 | No reliable identification | 1.661 | *A. nishimurae* | 1.834 | No reliable identification | 1.700 |
|  |  | *A. fumigatus* | 1.939 | *A. fumigatus* | 1.928 | *A. fumigatus* | 1.939 | *A. fumigatus* | 1.928 | *A. fumigatus* | 1.794 | *A. fumigatus* | 1.774 | *A. nishimurae* | 1.821 | *A. fumigatus* | 1.709 |
| DTO 310-D2 | *A. fumigatus* | *A. fumigatus* | 2.231 | *A. fumigatus* | 2.142 | *A. fumigatus* | 2.231 | *A. fumigatus* | 2.142 | *A. nishimurae* | 1.841 | *A. fumigatus* | 1.837 | *A. nishimurae* | 1.877 | *A. fumigatus* | 1.809 |
|  |  | No peaks found | 0 | No peaks found | 0 | No peaks found | 0 | No peaks found | 0 | No peaks found | 0 | No peaks found | 0 | No peaks found | 0 | No peaks found | 0 |
| DTO 310-D5 | *A. flavus* | *A. flavus* | 2.186 | *A. minisclerotigenes* | 2.181 | *A. flavus* | 2.186 | *A. minisclerotigenes* | 2.181 | *A. flavus* | 2.135 | *A. flavus* | 1.884 | *A. minisclerotigenes* | 2.130 | *A. flavus* | 2.114 |
|  |  | *A. flavus* | 2.508 | *A. flavus* | 2.479 | *A. flavus* | 2.350 | *A. flavus* | 2.323 | *A. flavus* | 2.452 | *A. flavus* | 2.445 | *A. flavus* | 2.508 | *A. flavus* | 2.479 |
| DTO 310-E5 | *A. tamarii* | *A. tamarii* | 2.579 | *A. tamarii* | 2.548 | *A. tamarii* | 2.258 | *A. tamarii* | 2.240 | *A. tamarii* | 2.579 | *A. tamarii* | 2.295 | *A. tamarii* | 2.548 | *A. tamarii* | 2.438 |
|  |  | *A. tamarii* | 2.467 | *A. tamarii* | 2.324 | *A. tamarii* | 2.131 | *A. tamarii* | 1.962 | *A. tamarii* | 2.324 | *A. cerealis* | 2.076 | *A. tamarii* | 2.467 | *A. mottae* | 1.999 |
| DTO 310-F9 | *A. flavus* | *A. pipericola* | 2.483 | *A. aflatoxiformans* | 2.482 | *A. oryzae* | 2.355 | *A. austwickii* | 2.302 | *A. pipericola* | 2.483 | *A. aflatoxiformans* | 2.482 | *A. aflatoxiformans* | 2.480 | *A. cerealis* | 2.344 |
|  |  | *A. pipericola* | 2.534 | *A. flavus* | 2.509 | *A. oryzae* | 2.340 | *A. oryzae* | 2.306 | *A. pipericola* | 2.534 | *A. flavus* | 2.509 | *A. flavus* | 2.419 | *A. aflatoxiformans* | 2.414 |
| DTO 310-G2 | *A. flavus* | *A. pipericola* | 2.568 | *A. flavus* | 2.535 | *A. flavus* | 2.348 | *A. austwickii* | 2.273 | *A. pipericola* | 2.568 | *A. flavus* | 2.535 | *A. flavus* | 2.504 | *A. aflatoxiformans* | 2.480 |
|  |  | *A. flavus* | 2.625 | *A. pipericola* | 2.594 | *A. flavus* | 2.335 | *A. flavus* | 2.319 | *A. flavus* | 2.625 | *A. pipericola* | 2.594 | *A. flavus* | 2.544 | *A. flavus* | 2.509 |
| DTO 310-G3 | *A. flavus* | *A. aflatoxiformans* | 2.537 | *A. aflatoxiformans* | 2.498 | *A. oryzae* | 2.275 | *A. austwickii* | 2.270 | *A. aflatoxiformans* | 2.537 | *A. pipericola* | 2.478 | *no peaks found* | 0 | No peaks found | 0 |
|  |  | No peaks found | 0 | No peaks found | 0 | No peaks found | 0 | No peaks found | 0 | No peaks found | 0 | No peaks found | 0 | *A. aflatoxiformans* | 2.498 | *A. minisclerotigenes* | 2.382 |
| DTO 310-G6 | *A. tamarii* | *A. tamarii* | 2.305 | *A. tamarii* | 2.247 | *A. togoensis* | 1.999 | *A. lanosus* | 1.930 | *A. tamarii* | 2.247 | *A. tamarii* | 2.229 | *A. tamarii* | 2.305 | *A. cerealis* | 2.140 |
|  |  | *A. tamarii* | 2.346 | *A. tamarii* | 2.223 | *A. aflatoxiformans* | 1.889 | *A. flavus* | 1.884 | *A. tamarii* | 2.223 | *A. pipericola* | 2.215 | *A. tamarii* | 2.346 | *A. cerealis* | 2.193 |
| DTO 310-G7 | *A. niger* | *A. niger* | 2.589 | *A. niger* | 2.573 | *A. niger* | 2.485 | *A. niger* | 2.366 | *A. niger* | 2.589 | *A. niger* | 2.573 | *A. tubingensis* | 2.467 | *A. tubingensis* | 2.400 |
|  |  | *A. niger* | 2.612 | *A. niger* | 2.576 | *A. niger* | 2.460 | *A. niger* | 2.391 | *A. niger* | 2.612 | *A. niger* | 2.576 | *A. tubingensis* | 2.375 | *A. tubingensis* | 2.359 |
| DTO 310-G8 | *A. flavus* | *A. flavus* | 2.550 | *A. pipericola* | 2.538 | *A. austwickii* | 2.297 | *A. novoparasiticus* | 2.284 | *A. flavus* | 2.550 | *A. pipericola* | 2.538 | *A. cerealis* | 2.470 | *A. flavus* | 2.454 |
|  |  | No peaks found | 0 | No peaks found | 0 | No peaks found | 0 | No peaks found | 0 | No peaks found | 0 | No peaks found | 0 | No peaks found | 0 | No peaks found | 0 |
| DTO 310-H2 | *A. flavus* | *A. minisclerotigenes* | 2.142 | *A. minisclerotigenes* | 2.011 | *A. minisclerotigenes* | 2.142 | *A. minisclerotigenes* | 2.011 | *A. flavus* | 1.869 | *A. cerealis* | 1.868 | *A. flavus* | 1.931 | *A. minisclerotigenes* | 1.923 |
|  |  | *A. flavus* | 2.096 | *A. flavus* | 2.093 | *A. flavus* | 2.040 | *A. flavus* | 1.973 | *A. flavus* | 2.093 | *A. flavus* | 2.034 | *A. flavus* | 2.096 | *A. flavus* | 2.070 |
| DTO 310-I6 | *A. flavus* | *A. pipericola* | 2.480 | *A. pipericola* | 2.471 | *A. oryzae* | 2.237 | *A. flavus* | 2.232 | *A. pipericola* | 2.480 | *A. aflatoxiformans* | 2.471 | *A. aflatoxiformans* | 2.452 | *A. minisclerotigenes* | 2.391 |
|  |  | No peaks found | 0 | No peaks found | 0 | No peaks found | 0 | No peaks found | 0 | No peaks found | 0 | No peaks found | 0 | No peaks found | 0 | No peaks found | 0 |
| DTO 310-I7 | *A. flavus* | *A. pipericola* | 2.549 | *A. pipericola* | 2.479 | *A. flavus* | 2.342 | *A. minisclerotigenes* | 2.283 | *A. pipericola* | 2.549 | *A. aflatoxiformans* | 2.479 | *A. cerealis* | 2.435 | *A. aflatoxiformans* | 2.387 |
|  |  | No peaks found | 0 | No peaks found | 0 | No peaks found | 0 | No peaks found | 0 | No peaks found | 0 | No peaks found | 0 | No peaks found | 0 | No peaks found | 0 |
| DTO 310-I8 | *A. flavus* | *A. pipericola* | 2.511 | *A. flavus* | 2.505 | *A. austwickii* | 2.360 | *A. flavus* | 2.341 | *A. pipericola* | 2.511 | *A. cerealis* | 2.505 | *A. aflatoxiformans* | 2.452 | *A. flavus* | 2.449 |
|  |  | *A. flavus* | 2.574 | *A. flavus* | 2.517 | *A. austwickii* | 2.396 | *A. flavus* | 2.312 | *A. flavus* | 2.574 | *A. flavus* | 2.517 | *A. flavus* | 2.475 | *A. flavus* | 2.429 |
| DTO 310-I9 | *A. welwitschiae* | *A. niger* | 2.642 | *A. tubingensis* | 2.415 | *A. niger* | 2.370 | *A. tubingensis* | 2.279 | *A. niger* | 2.642 | *A. tubingensis* | 2.415 | *A. tubingensis* | 2.384 | *A. tubingensis* | 2.320 |
|  |  | *A. niger* | 2.617 | *A. tubingensis* | 2.518 | *A. tubingensis* | 2.438 | *A. tubingensis* | 2.424 | *A. niger* | 2.617 | *A. tubingensis* | 2.479 | *A. tubingensis* | 2.518 | *A. tubingensis* | 2.409 |
| DTO 311-A1 | *A. flavus* | *A. flavus* | 1.988 | *A. parasiticus* | 1.975 | *A. flavus* | 2.367 | *A. flavus* | 2.310 | *A. flavus* | 1.988 | *A. flavus* | 1.970 | *A. flavus* | 1.900 | *A. aflatoxiformans* | 1.897 |
|  |  | *A. flavus* | 2.554 | *A. flavus* | 2.528 | *A. parasiticus* | 1.975 | *A. cerealis* | 1.962 | *A. flavus* | 2.554 | *A. flavus* | 2.528 | *A. flavus* | 2.468 | *A. aflatoxiformans* | 2.446 |
| DTO 311-A2 | *A. aff. niger* | *A. niger* | 2.520 | *A. niger* | 2.473 | *A. niger* | 2.456 | *A. niger* | 2.377 | *A. niger* | 2.520 | *A. niger* | 2.473 | *A. niger* | 2.434 | *A. tubingensis* | 2.404 |
|  |  | *A. niger* | 2.578 | *A. niger* | 2.576 | *A. niger* | 2.501 | *A. tubingensis* | 2.479 | *A. niger* | 2.578 | *A. niger* | 2.576 | *A. niger* | 2.475 | *A. niger* | 2.436 |
| DTO 311-A4 | *A. niger* | *A. niger* | 2.567 | *A. niger* | 2.519 | *A. niger* | 2.519 | *A. niger* | 2.416 | *A. niger* | 2.567 | *A. niger* | 2.426 | *A. tubingensis* | 2.375 | *A. tubingensis* | 2.361 |
|  |  | *A. niger* | 2.588 | *A. niger* | 2.549 | *A. niger* | 2.466 | *A. tubingensis* | 2.418 | *A. niger* | 2.588 | *A. niger* | 0 | *A. tubingensis* | 2.520 | *A. tubingensis* | 2.393 |
| DTO 311-A5 | *A. flavus* | *A. flavus* | 2.538 | *A. flavus* | 2.526 | *A. oryzae* | 2.277 | *A. novoparasiticus* | 2.210 | *A. flavus* | 2.538 | *A. flavus* | 2.526 | *A. flavus* | 2.485 | *A. cerealis* | 2.449 |
|  |  | *A. pipericola* | 2.570 | *A. flavus* | 2.515 | *A. novoparasiticus* | 2.225 | *A. novoparasiticus* | 2.219 | *A. pipericola* | 2.570 | *A. flavus* | 2.515 | *A. flavus* | 2.475 | *A. cerealis* | 2.461 |
| DTO 311-A6 | *A. flavus* | *A. flavus* | 2.529 | *A. flavus* | 2.521 | *A. austwickii* | 2.346 | *A. flavus* | 2.281 | *A. flavus* | 2.529 | *A. flavus* | 2.521 | *A. aflatoxiformans* | 2.483 | *A. flavus* | 2.472 |
|  |  | No peaks found | 0 | No peaks found | 0 | No peaks found | 0 | No peaks found | 0 | No peaks found | 0 | No peaks found | 0 | No peaks found | 0 | No peaks found | 0 |
| DTO 311-A8 | *A. flavus* | *A. flavus* | 2.472 | *A. flavus* | 2.470 | *A. flavus* | 2.322 | *A. flavus* | 2.321 | *A. flavus* | 2.472 | *A. flavus* | 2.449 | *A. flavus* | 2.470 | *A. flavus* | 2.44 |
|  |  | *A. pipericola* | 2.538 | *A. flavus* | 2.538 | *A. austwickii* | 2.445 | *A. flavus* | 2.360 | *A. pipericola* | 2.538 | *A. flavus* | 2.538 | *A. flavus* | 2.524 | *A. aflatoxiformans* | 2.46 |
| DTO 311-B4 | *A. welwitschiae* | *A. niger* | 2.554 | *A. tubingensis* | 2.548 | *A. tubingensis* | 2.480 | *A. niger* | 2.472 | *A. niger* | 2.554 | *A. tubingensis* | 2.548 | *A. tubingensis* | 2.532 | *A. tubingensis* | 2.491 |
|  |  | No peaks found | 0 | No peaks found | 0 | No peaks found | 0 | No peaks found | 0 | No peaks found | 0 | No peaks found | 0 | No peaks found | 0 | No peaks found | 0 |
| DTO 311-B6 | *A. welwitschiae* | *A. niger* | 2.615 | *A. tubingensis* | 2.577 | *A. tubingensis* | 2.469 | *A. niger* | 2.454 | *A. niger* | 2.615 | *A. tubingensis* | 2.560 | *A. tubingensis* | 2.577 | *A. tubingensis* | 2.472 |
|  |  | *A. niger* | 2.588 | *A. niger* | 2.492 | *A. tubingensis* | 2.428 | *A. niger* | 2.374 | *A. niger* | 2.588 | *A. niger* | 2.492 | *A. tubingensis* | 2.487 | *A. tubingensis* | 2.483 |
| DTO 311-B9 | *A. welwitschiae* | *A. niger* | 2.490 | *A. niger* | 2.395 | *A. niger* | 2.395 | *A. tubingensis* | 2.271 | *A. niger* | 2.490 | *A. niger* | 2.360 | *A. niger* | 2.166 | *A. niger* | 2.151 |
|  |  | *A. niger* | 2.365 | *A. niger* | 2.308 | *A. niger* | 2.365 | *A. niger* | 2.173 | *A. niger* | 2.308 | *A. niger* | 2.243 | *A. niger* | 2.206 | *A. tubingensis* | 2.032 |
| DTO 311-C1 | *A. flavus* | *A. flavus* | 2.566 | *A. pipericola* | 2.522 | *A. flavus* | 2.371 | *A. austwickii* | 2.365 | *A. flavus* | 2.566 | *A. pipericola* | 2.522 | *A. aflatoxiformans* | 2.438 | *A. flavus* | 2.434 |
|  |  | *A. flavus* | 2.614 | *A. flavus* | 2.547 | *A. flavus* | 2.430 | *A. austwickii* | 2.378 | *A. flavus* | 2.614 | *A. flavus* | 2.547 | *A. flavus* | 2.518 | *A. flavus* | 2.478 |
| DTO 311-C2 | *A. flavus* | *A. flavus* | 2.614 | *A. flavus* | 2.598 | *A. austwickii* | 2.406 | *A. flavus* | 2.364 | *A. flavus* | 2.614 | *A. flavus* | 2.598 | *A. flavus* | 2.539 | *A. aflatoxiformans* | 2.501 |
|  |  | *A. flavus* | 2.592 | *A. flavus* | 2.581 | *A. austwickii* | 2.451 | *A. flavus* | 2.366 | *A. flavus* | 2.592 | *A. flavus* | 2.581 | *A. flavus* | 2.560 | *A. aflatoxiformans* | 2.520 |
| DTO 311-C4 | *A. flavus* | *A. flavus* | 2.607 | *A. flavus* | 2.578 | *A. austwickii* | 2.327 | *A. oryzae* | 2.230 | *A. flavus* | 2.607 | *A. flavus* | 2.578 | *A. cerealis* | 2.526 | *A. flavus* | 2.497 |
|  |  | *A. flavus* | 2.531 | *A. pipericola* | 2.527 | *A. novoparasiticus* | 2.293 | *A. austwickii* | 2.282 | *A. flavus* | 2.531 | *A. pipericola* | 2.527 | *A. flavus* | 2.429 | *A. aflatoxiformans* | 2.424 |
| DTO 311-C7 | *A. tamarii* | *A. tamarii* | 2.449 | *A. tamarii* | 2.340 | *A. austwickii* | 2.013 | *A. flavus* | 1.936 | *A. tamarii* | 2.340 | *A. pipericola* | 2.238 | *A. tamarii* | 2.449 | *A. cerealis* | 2.144 |
|  |  | *A. tamarii* | 2.392 | *A. tamarii* | 2.349 | *A. novoparasiticus* | 1.999 | *A. cerealis* | 1.931 | *A. tamarii* | 2.349 | *A. novoparasiticus* | 2.179 | *A. tamarii* | 2.392 | *A. cerealis* | 2.180 |
| DTO 311-C8 | *A. flavus* | No peaks found | 0 | No peaks found | 0 | No peaks found | 0 | No peaks found | 0 | No peaks found | 0 | No peaks found | 0 | No peaks found | 0 | No peaks found | 0 |
|  |  | *A. flavus* | 2.484 | *A. flavus* | 2.459 | *A. flavus* | 2.327 | *A. austwickii* | 2.304 | *A. flavus* | 2.484 | *A. flavus* | 2.459 | *A. flavus* | 2.428 | *A. cerealis* | 2.404 |
| DTO 311-D3 | *A. flavus* | *A. flavus* | 2.519 | *A. transmontanensis* | 2.488 | *A. austwickii* | 2.310 | *A. oryzae* | 2.296 | *A. flavus* | 2.519 | *A. transmo* | 2.488 | *A. sojae* | 2.447 | *A. parasiticus-soja* | 2.416 |
|  |  | *A. flavus* | 2.470 | *A. aflatoxiformans* | 2.457 | *A. austwickii* | 2.308 | *A. flavus* | 2.219 | *A. flavus* | 2.470 | *A. pipericola* | 2.411 | *A. aflatoxiformans* | 2.457 | *A. flavus* | 2.373 |
| DTO 311-F8 | *A. flavus* | *A. flavus* | 2.572 | *A. pipericola* | 2.528 | *A. flavus* | 2.430 | *A. oryzae* | 2.373 | *A. flavus* | 2.572 | *A. pipericola* | 2.528 | *A. cerealis* | 2.469 | *A. aflatoxiformans* | 2.445 |
|  |  | *A. flavus* | 2.657 | *A. flavus* | 2.597 | *A. oryzae* | 2.346 | *A. flavus* | 2.279 | *A. flavus* | 2.657 | *A. flavus* | 2.597 | *A. flavus* | 2.591 | *A. flavus* | 2.518 |
| DTO 311-G3 | *A. flavus* | *A. pipericola* | 2.553 | *A. aflatoxiformans* | 2.496 | *A. flavus* | 2.322 | *A. flavus* | 2.283 | *A. pipericola* | 2.553 | *A. aflatoxiformans* | 2.496 | *A. cerealis* | 2.478 | *A. aflatoxiformans* | 2.430 |
|  |  | *A. pipericola* | 2.544 | *A. aflatoxiformans* | 2.492 | *A. austwickii* | 2.388 | *A. flavus* | 2.330 | *A. pipericola* | 2.544 | *A. aflatoxiformans* | 2.492 | *A. aflatoxiformans* | 2.469 | *A. cerealis* | 2.447 |
| DTO 310-A7 | *A. flavus* | *A. flavus* | 2.600 | *A. flavus* | 2.583 | *A. flavus* | 2.435 | *A. flavus* | 2.421 | *A. flavus* | 2.550 | *A. flavus* | 2.535 | *A. flavus* | 2.600 | *A. flavus* | 2.583 |
|  |  | *A. flavus* | 2.607 | *A. flavus* | 2.606 | *A. flavus* | 2.382 | *A. flavus* | 2.371 | *A. flavus* | 2.592 | *A. flavus* | 2.542 | *A. flavus* | 2.607 | *A. flavus* | 2.606 |
| DTO 310-A9 | *A. flavus* | *A. flavus* | 2.663 | *A. flavus* | 2.652 | *A. flavus* | 2.329 | *A. minisclerotigenes* | 2.310 | *A. flavus* | 2.652 | *A. flavus* | 2.629 | *A. flavus* | 2.663 | *A. flavus* | 2.620 |
|  |  | *A. flavus* | 2.570 | *A. flavus* | 2.548 | *A. oryzae* | 2.281 | *A. minisclerotigenes* | 2.245 | *A. flavus* | 2.570 | *A. flavus* | 2.522 | *A. flavus* | 2.548 | *A. flavus* | 2.529 |
| DTO 310-B2 | *A. flavus* | *A. flavus* | 2.570 | *A. flavus* | 2.537 | *A. flavus* | 2.337 | *A. flavus* | 2.313 | *A. flavus* | 2.499 | *A. flavus* | 2.476 | *A. flavus* | 2.570 | *A. flavus* | 2.537 |
|  |  | *A. flavus* | 2.611 | *A. flavus* | 2.584 | *A. flavus* | 2.337 | *A. flavus* | 2.299 | *A. flavus* | 2.584 | *A. flavus* | 2.553 | *A. flavus* | 2.611 | *A. flavus* | 2.553 |
| DTO 310-B4 | *A. flavus* | *A. flavus* | 2.582 | *A. flavus* | 2.564 | *A. flavus* | 2.339 | *A. flavus* | 2.271 | *A. flavus* | 2.541 | *A. flavus* | 2.498 | *A. flavus* | 2.582 | *A. flavus* | 2.564 |
|  |  | *A. flavus* | 2.518 | *A. flavus* | 2.447 | *A. flavus* | 2.168 | *A. minisclerotigenes* | 2.165 | *A. flavus* | 2.442 | *A. flavus* | 2.409 | *A. flavus* | 2.518 | *A. flavus* | 2.447 |
| DTO 310-B5 | *A. fumigatus* | *A. fumigatus* | 2.375 | *A. fumigatus* | 2.351 | *A. fumigatus* | 2.375 | *A. fumigatus* | 2.342 | *A. fumigatus* | 2.248 | *A. fumigatus* | 2.238 | *A. fumigatus* | 2.351 | *A. fumigatus* | 2.325 |
|  |  | *A. fumigatus* | 2.429 | *A. fumigatus* | 2.347 | *A. fumigatus* | 2.429 | *A. fumigatus* | 2.347 | *A. fumigatus* | 2.134 | *A. fumigatus* | 2.128 | *A. fumigatus* | 2.272 | *A. fumigatus* | 2.225 |
| DTO 310-B6 | *A. fumigatus* | *A. fumigatus* | 2.191 | *A. fumigatus* | 2.168 | *A. fumigatus* | 2.191 | *A. fumigatus* | 2.168 | *A. fumigatus* | 1.952 | *A. fumigatus* | 1.877 | *A. fumigatus* | 2.090 | *A. fumigatus* | 1.841 |
|  |  | *A. fumigatus* | 2.355 | *A. fumigatus* | 2.303 | *A. fumigatus* | 2.355 | *A. fumigatus* | 2.303 | *A. fumigatus* | 2.005 | *A. fumigatus* | 1.906 | *A. fumigatus* | 2.050 | *A. fumigatus* | 1.874 |
| DTO 310-B7 | *Pen. rubens* | *Pen. chrysogenum* | 2.059 | *P. chrysogenum* | 2.041 | *P. chrysogenum* | 2.059 | *P. chrysogenum* | 2.041 | No reliable identification | 1.033 | No reliable identification | 0.908 | No reliable identification | 0.915 | No reliable identification | 0.914 |
|  |  | *Pen. chrysogenum* | 2.084 | *P. chrysogenum* | 2.070 | *P. chrysogenum* | 2.084 | *P. chrysogenum* | 2.070 | No reliable identification | 1.211 | No reliable identification | 1.210 | No reliable identification | 1.170 | No reliable identification | 1.044 |
| DTO 310-B8 | *A. fumigatus* | *A. fumigatus* | 2.352 | *A. fumigatus* | 2.325 | *A. fumigatus* | 2.352 | *A. fumigatus* | 2.325 | *A. fumigatus* | 2.248 | *A. fumigatus* | 2.127 | *A. fumigatus* | 2.223 | *A. fumigatus* | 2.155 |
|  |  | *A. fumigatus* | 2.240 | *A. fumigatus* | 2.122 | *A. fumigatus* | 2.240 | *A. fumigatus* | 2.122 | *A. nishimurae* | 2.052 | *A. fumigatus* | 2.050 | *A. fumigatus* | 2.041 | *A. fumigatus* | 2.032 |
| DTO 310-B9 | *A. flavus* | *A. flavus* | 2.582 | *A. flavus* | 2.536 | *A. flavus* | 2.255 | *A. aflatoxiformans* | 2.153 | *A. flavus* | 2.535 | *A. flavus* | 2.450 | *A. flavus* | 2.582 | *A. flavus* | 2.536 |
|  |  | *A. flavus* | 2.607 | *A. flavus* | 2.597 | *A. flavus* | 2.246 | *A. flavus* | 2.239 | *A. flavus* | 2.597 | *A. flavus* | 2.589 | *A. flavus* | 2.607 | *A. flavus* | 2.593 |
| DTO 310-C1 | *A. fumigatus* | No peaks found | 0 | No peaks found | 0 | No peaks found | 0 | No peaks found | 0 | No peaks found | 0 | No peaks found | 0 | No peaks found | 0 | No peaks found | 0 |
|  |  | No peaks found | 0 | No peaks found | 0 | No peaks found | 0 | No peaks found | 0 | No peaks found | 0 | No peaks found | 0 | No peaks found | 0 | No peaks found | 0 |
| DTO 310-C2 | *A. flavus* | *A. flavus* | 2.582 | *A. flavus* | 2.576 | *A. flavus* | 2.348 | *A. oryzae* | 2.302 | *A. flavus* | 2.576 | *A. flavus* | 2.576 | *A. flavus* | 2.582 | *A. flavus* | 2.534 |
|  |  | *A. flavus* | 2.568 | *A. flavus* | 2.563 | *A. flavus* | 2.270 | *A. oryzae* | 2.243 | *A. flavus* | 2.568 | *A. flavus* | 2.539 | *A. flavus* | 2.563 | *A. flavus* | 2.518 |
| DTO 310-C3 | *A. flavus* | *A. flavus* | 2.598 | *A. flavus* | 2.563 | *A. flavus* | 2.385 | *A. flavus* | 2.312 | *A. flavus* | 2.513 | *A. flavus* | 2.501 | *A. flavus* | 2.598 | *A. flavus* | 2.563 |
|  |  | *A. flavus* | 2.559 | *A. flavus* | 2.531 | *A. flavus* | 2.283 | *A. flavus* | 2.208 | *A. flavus* | 2.559 | *A. flavus* | 2.472 | *A. flavus* | 2.531 | *A. flavus* | 2.500 |
| DTO 310-C4 | *A. flavus* | *A. flavus* | 2.380 | *A. flavus* | 2.342 | *A. flavus* | 2.206 | *A. flavus* | 2.088 | *A. flavus* | 2.342 | *A. flavus* | 2.329 | *A. flavus* | 2.380 | *A. flavus* | 2.314 |
|  |  | *A. flavus* | 2.573 | *A. flavus* | 2.569 | *A. flavus* | 2.268 | *A. flavus* | 2.267 | *A. flavus* | 2.561 | *A. flavus* | 2.561 | *A. flavus* | 2.573 | *A. flavus* | 2.569 |
| DTO 310-C5 | *A. flavus* | *A. minisclerotigenes* | 2.170 | *A. minisclerotigenes* | 2.096 | *A. minisclerotigenes* | 2.170 | *A. minisclerotigenes* | 2.096 | *A. flavus* | 1.795 | No reliable identification | 1.697 | *A. flavus* | 1.890 | *A. flavus* | 1.842 |
|  |  | *A. minisclerotigenes* | 2.146 | *A. flavus* | 2.062 | *A. minisclerotigenes* | 2.146 | *A. flavus* | 2.062 | *A. flavus* | 1.880 | *A. flavus* | 1.824 | *A. flavus* | 1.897 | *A. flavus* | 1.891 |
| DTO 310-C6 | *A. flavus* | *A. flavus* | 2.524 | *A. flavus* | 2.523 | *A. oryzae* | 2.388 | *A. flavus* | 2.372 | *A. flavus* | 2.524 | *A. flavus* | 2.434 | *A. flavus* | 2.523 | *A. flavus* | 2.427 |
|  |  | *A. flavus* | 2.517 | *A. flavus* | 2.497 | *A. oryzae* | 2.401 | *A. flavus* | 2.399 | *A. flavus* | 2.517 | *A. flavus* | 2.483 | *A. flavus* | 2.497 | *A. flavus* | 2.442 |
| DTO 310-C7 | *A. flavus* | *A. flavus* | 2.576 | *A. flavus* | 2.560 | *A. flavus* | 2.247 | *A. austwickii* | 2.203 | *A. flavus* | 2.560 | *A. flavus* | 2.550 | *A. flavus* | 2.576 | *A. flavus* | 2.554 |
|  |  | *A. flavus* | 2.672 | *A. flavus* | 2.633 | *A. flavus* | 2.369 | *A. oryzae* | 2.292 | *A. flavus* | 2.614 | *A. flavus* | 2.598 | *A. flavus* | 2.672 | *A. flavus* | 2.633 |
| DTO 310-C8 | *A. flavus* | *A. flavus* | 2.509 | *A. flavus* | 2.506 | *A. flavus* | 2.344 | *A. flavus* | 2.287 | *A. flavus* | 2.509 | *A. flavus* | 2.448 | *A. flavus* | 2.506 | *A. flavus* | 2.471 |
|  |  | *A. flavus* | 2.587 | *A. flavus* | 2.584 | *A. flavus* | 2.349 | *A. flavus* | 2.328 | *A. flavus* | 2.480 | *A. flavus* | 2.473 | *A. flavus* | 2.587 | *A. flavus* | 2.584 |
| DTO 310-C9 | *A. flavus* | *A. flavus* | 2.508 | *A. flavus* | 2.502 | *A. flavus* | 2.338 | *A. minisclerotigenes* | 2.328 | *A. flavus* | 2.508 | *A. flavus* | 2.463 | *A. flavus* | 2.502 | *A. flavus* | 2.442 |
|  |  | *A. flavus* | 2.537 | *A. flavus* | 2.494 | *A. flavus* | 2.338 | *A. oryzae* | 2.298 | *A. flavus* | 2.494 | *A. flavus* | 2.460 | *A. flavus* | 2.537 | *A. flavus* | 2.489 |
| DTO 310-D1 | *A. flavus* | *A. flavus* | 2.413 | *A. flavus* | 2.387 | *A. flavus* | 2.387 | *A. flavus* | 2.225 | *A. flavus* | 2.324 | *A. flavus* | 2.280 | *A. flavus* | 2.413 | *A. flavus* | 2.375 |
|  |  | No peaks found | 0 | No peaks found | 0 | No peaks found | 0 | No peaks found | 0 | No peaks found | 0 | No peaks found | 0 | No peaks found | 0 | No peaks found | 0 |
| DTO 310-D3 | *A. flavus* | *A. flavus* | 2.481 | *A. flavus* | 2.466 | *A. flavus* | 2.287 | *A. oryzae* | 2.264 | *A. flavus* | 2.481 | *A. flavus* | 2.412 | *A. flavus* | 2.466 | *A. flavus* | 2.424 |
|  |  | *A. flavus* | 2.396 | *A. flavus* | 2.384 | *A. oryzae* | 2.319 | *A. oryzae* | 2.319 | *A. flavus* | 2.396 | *A. flavus* | 2.384 | *A. flavus* | 2.359 | *A. flavus* | 2.325 |
| DTO 310-D4 | *A. fumigatus* | *A. fumigatus* | 2.120 | *A. fumigatus* | 2.114 | *A. fumigatus* | 2.120 | *A. fumigatus* | 2.114 | *A. fumigatus* | 1.834 | *A. fumigatus* | 1.794 | *A. fumigatus* | 1.911 | *A. fumigatus* | 1.906 |
|  |  | No reliable identification | 1.548 | No reliable identification | 1.353 | No reliable identification | 1.353 | No reliable identification | 1.284 | No reliable identification | 1.329 | No reliable identification | 1.237 | No reliable identification | 1.548 | No reliable identification | 1.219 |
| DTO 310-D6 | *A. terreus* | *A. terreus* | 2.455 | *A. terreus* | 2.451 | *A. terreus* | 2.455 | *A. terreus* | 2.451 | No reliable identification | 1.423 | No reliable identification | 1.399 | No reliable identification | 1.472 | No reliable identification | 1.374 |
|  |  | *A. terreus* | 2.432 | *A. terreus* | 2.429 | *A. terreus* | 2.432 | *A. terreus* | 2.429 | No reliable identification | 1.601 | No reliable identification | 1.555 | No reliable identification | 1.465 | No reliable identification | 1.460 |
| DTO 310-D7 | *A. flavus* | *A. flavus* | 2.495 | *A. flavus* | 2.423 | *A. oryzae* | 2.131 | *A. flavus* | 2.116 | *A. flavus* | 2.495 | *A. flavus* | 2.423 | *A. flavus* | 2.403 | *A. flavus* | 2.363 |
|  |  | *A. flavus* | 2.402 | *A. flavus* | 2.333 | *A. oryzae* | 2.120 | *A. oryzae* | 2.096 | *A. flavus* | 2.402 | *A. flavus* | 2.333 | *A. flavus* | 2.296 | *A. flavus* | 2.210 |
| DTO 310-D8 | *A. fumigatus* | *A. fumigatus* | 2.431 | *A. fumigatus* | 2.382 | *A. fumigatus* | 2.431 | *A. fumigatus* | 2.382 | *A. fumigatus* | 2.160 | *A. fumigatus* | 2.057 | *A. fumigatus* | 2.156 | *A. fumigatus* | 2.102 |
|  |  | *A. fumigatus* | 2.431 | *A. fumigatus* | 2.348 | *A. fumigatus* | 2.431 | *A. fumigatus* | 2.348 | *A. fumigatus* | 2.231 | *A. fumigatus* | 2.134 | *A. fumigatus* | 2.202 | *A. fumigatus* | 2.133 |
| DTO 310-D9 | *A. neoniger* | *A. tubingensis* | 2.663 | *A. tubingensis* | 2.641 | *A. tubingensis* | 2.578 | *A. tubingensis* | 2.526 | *A. tubingensis* | 2.663 | *A. tubingensis* | 2.641 | *A. tubingensis* | 2.561 | *A. tubingensis* | 2.558 |
|  |  | *A. tubingensis* | 2.364 | *A. tubingensis* | 2.364 | *A. tubingensis* | 2.332 | *A. tubingensis* | 2.288 | *A. tubingensis* | 2.364 | *A. tubingensis* | 2.364 | *A. tubingensis* | 2.163 | *A. tubingensis* | 2.161 |
| DTO 310-E1 | *A. flavus* | *A. flavus* | 2.611 | *A. flavus* | 2.597 | *A. flavus* | 2.397 | *A. flavus* | 2.379 | *A. flavus* | 2.597 | *A. pipericola* | 2.489 | *A. flavus* | 2.611 | *A. flavus* | 2.517 |
|  |  | *A. flavus* | 2.692 | *A. flavus* | 2.621 | *A. flavus* | 2.418 | *A. flavus* | 2.412 | *A. flavus* | 2.621 | *A. pipericola* | 2.524 | *A. flavus* | 2.692 | *A. flavus* | 2.599 |
| DTO 310-E2 | *A. welwitschiae* | *A. niger* | 2.581 | *A. niger* | 2.512 | *A. niger* | 2.414 | *A. niger* | 2.393 | *A. niger* | 2.581 | *A. niger* | 2.512 | *A. niger* | 2.454 | *A. niger* | 2.446 |
|  |  | *A. niger* | 2.591 | *A. niger* | 2.548 | *A. niger* | 2.395 | *A. niger* | 2.384 | *A. niger* | 2.591 | *A. niger* | 2.548 | *A. niger* | 2.525 | *A. niger* | 2.480 |
| DTO 310-E3 | *Aspergilus niger* | *A. niger* | 2.152 | *A. niger* | 2.106 | *A. niger* | 1.990 | *A. tubingensis* | 1.876 | *A. niger* | 2.106 | *A. niger* | 1.963 | *A. niger* | 2.152 | *A. niger* | 1.892 |
|  |  | *A. niger* | 2.603 | *A. niger* | 2.590 | *A. niger* | 2.444 | *A. niger* | 2.433 | *A. niger* | 2.603 | *A. niger* | 2.590 | *A. niger* | 2.470 | *A. niger* | 2.460 |
| DTO 310-E4 | *A. fumigatus* | *A. fumigatus* | 2.318 | *A. fumigatus* | 2.298 | *A. fumigatus* | 2.296 | *A. fumigatus* | 2.235 | *A. fumigatus* | 2.298 | *A. fumigatus* | 2.229 | *A. fumigatus* | 2.318 | *A. fumigatus* | 2.194 |
|  |  | *A. fumigatus* | 2.295 | *A. fumigatus* | 2.185 | *A. fumigatus* | 2.295 | *A. fumigatus* | 2.183 | *A. fumigatus* | 2.185 | *A. fumigatus* | 2.134 | *A. fumigatus* | 2.062 | *A. fumigatus* | 1.996 |
| DTO 310-E6 | *A. flavus* | *A. flavus* | 2.627 | *A. flavus* | 2.567 | *A. flavus* | 2.402 | *A. flavus* | 2.382 | *A. flavus* | 2.448 | *A. flavus* | 2.446 | *A. flavus* | 2.627 | *A. flavus* | 2.567 |
|  |  | *A. flavus* | 2.302 | *A. flavus* | 2.209 | *A. flavus* | 2.168 | *A. flavus* | 2.103 | *A. flavus* | 2.161 | *A. flavus* | 2.145 | *A. flavus* | 2.302 | *A. flavus* | 2.209 |
| DTO 310-E7 | *A. flavus* | *A. flavus* | 2.617 | *A. flavus* | 2.617 | *A. flavus* | 2.340 | *A. oryzae* | 2.312 | *A. flavus* | 2.617 | *A. flavus* | 2.617 | *A. flavus* | 2.612 | *A. flavus* | 2.612 |
|  |  | *A. flavus* | 2.632 | *A. flavus* | 2.584 | *A. aflatoxiformans* | 2.333 | *A. flavus* | 2.316 | *A. flavus* | 2.580 | *A. flavus* | 2.558 | *A. flavus* | 2.632 | *A. flavus* | 2.584 |
| DTO 310-E8 | *A. neoniger* | *A. tubigensis* | 2.359 | *A. tubingensis* | 2.351 | *A. tubingensis* | 2.351 | *A. tubingensis* | 2.336 | *A. tubingensis* | 2.359 | *A. tubingensis* | 2.324 | *A. tubingensis* | 2.261 | *A. tubingensis* | 2.228 |
|  |  | *A. tubigensis* | 2.669 | *A. tubingensis* | 2.632 | *A. tubingensis* | 2.547 | *A. tubingensis* | 2.530 | *A. tubingensis* | 2.669 | *A. tubingensis* | 2.632 | *A. tubingensis* | 2.618 | *A. tubingensis* | 2.575 |
| DTO 310-E9 | *A. neoniger* | *A. tubigensis* | 2.307 | *A. tubingensis* | 2.304 | *A. tubingensis* | 2.288 | *A. tubingensis* | 2.285 | *A. tubingensis* | 2.307 | *A. tubingensis* | 2.304 | *A. tubingensis* | 2.268 | *A. tubingensis* | 2.181 |
|  |  | *A. tubigensis* | 2.616 | *A. tubingensis* | 2.560 | *A. tubingensis* | 2.518 | *A. tubingensis* | 2.479 | *A. tubingensis* | 2.616 | *A. tubingensis* | 2.560 | *A. tubingensis* | 2.538 | *A. tubingensis* | 2.496 |
| DTO 310-F2 | *A. welwitschiae* | *A. niger* | 2.418 | *A. niger* | 2.380 | *A. niger* | 2.312 | *A. niger* | 2.200 | *A. niger* | 2.418 | *A. niger* | 2.380 | *A. niger* | 2.279 | *A. niger* | 2.277 |
|  |  | *A. niger* | 2.545 | *A. niger* | 2.517 | *A. niger* | 2.354 | *A. niger* | 2.351 | *A. niger* | 2.545 | *A. niger* | 2.517 | *A. niger* | 2.420 | *A. niger* | 2.393 |
| DTO 310-F3 | *A. niger* | *A. niger* | 2.575 | *A. niger* | 2.536 | *A. niger* | 2.381 | *A. niger* | 2.350 | *A. niger* | 2.575 | *A. niger* | 2.536 | *A. niger* | 2.502 | *A. niger* | 2.439 |
|  |  | *A. niger* | 2.472 | *A. niger* | 2.438 | *A. niger* | 2.401 | *A. niger* | 2.370 | *A. niger* | 2.472 | *A. niger* | 2.438 | *A. niger* | 2.324 | *A. niger* | 2.322 |
| DTO 310-F4 | *A. tamarii* | *A. tamarii* | 2.546 | *A. tamarii* | 2.470 | *A. tamarii* | 2.228 | *A. tamarii* | 2.192 | *A. tamarii* | 2.470 | *A. tamarii* | 2.267 | *A. tamarii* | 2.546 | *A. tamarii* | 2.292 |
|  |  | *A. tamarii* | 2.488 | *A. tamarii* | 2.326 | *A. tamarii* | 2.064 | *A. tamarii* | 1.911 | *A. tamarii* | 2.326 | *A. tamarii* | 2.258 | *A. tamarii* | 2.488 | *A. cerealis* | 2.141 |
| DTO 310-F5 | *P. citrinum* | *P. citrinum* | 2.086 | *P. citrinum* | 2.032 | *P. citrinum* | 2.086 | *P. citrinum* | 2.032 | No reliable identification | 1.180 | No reliable identification | 1.167 | No reliable identification | 1.257 | No reliable identification | 1.233 |
|  |  | *P. citrinum* | 2.017 | *P. citrinum* | 1.986 | *P. citrinum* | 2.017 | *P. citrinum* | 1.986 | No reliable identification | 1.183 | No reliable identification | 1.180 | No reliable identification | 1.232 | No reliable identification | 1.211 |
| DTO 310-F6 | *A. welwitschiae* | *A. niger* | 2.376 | *A. niger* | 2.321 | *A. tubingensis* | 2.200 | *A. tubingensis* | 2.107 | *A. niger* | 2.376 | *A. niger* | 2.321 | *A. niger* | 2.208 | *A. niger* | 2.181 |
|  |  | *A. niger* | 2.266 | *A. niger* | 2.235 | *A. tubingensis* | 2.115 | *A. niger* | 2.108 | *A. niger* | 2.266 | *A. niger* | 2.217 | *A. niger* | 2.235 | *A. niger* | 2.192 |
| DTO 310-F8 | *A. fumigatus* | *A. fumigatus* | 2.406 | *A. fumigatus* | 2.326 | *A. fumigatus* | 2.406 | *A. fumigatus* | 2.326 | *A. fumigatu* | 2.092 | *A. fumigatu* | 1.979 | *A. fumigatus* | 2.197 | *A. fumigatus* | 1.923 |
|  |  | *A. fumigatus* | 2.447 | *A. fumigatus* | 2.362 | *A. fumigatus* | 2.447 | *A. fumigatus* | 2.362 | *A. fumigatu* | 2.031 | *A. fumigatu* | 2.018 | *A. fumigatus* | 2.152 | *A. fumigatus* | 1.954 |
| DTO 310-G1 | *A. aculeatinus* | No reliable identification | 1.492 | No reliable identification | 1.457 | No reliable identification | 1.457 | No reliable identification | 1.362 | No reliable identification | 1.443 | No reliable identification | 1.401 | No reliable identification | 1.492 | No reliable identification | 1.394 |
|  |  | No reliable identification | 1.486 | No reliable identification | 1.416 | No reliable identification | 1.344 | No reliable identification | 1.342 | No reliable identification | 1.486 | No reliable identification | 1.286 | No reliable identification | 1.416 | No reliable identification | 1.408 |
| DTO 310-G4 | *A. fumigatus* | *A. fumigatus* | 2.208 | *A. fumigatus* | 2.130 | *A. fumigatus* | 2.208 | *A. fumigatus* | 2.122 | *A. fumigatus* | 2.130 | *A. fumigatu* | 2.068 | *A. fumigatus* | 2.125 | *A. fumigatus* | 1.954 |
|  |  | *A. fumigatus* | 2.294 | *A. fumigatus* | 2.233 | *A. fumigatus* | 2.294 | *A. fumigatus* | 2.175 | *A. fumigatus* | 2.233 | *A. fumigatu* | 2.156 | *A. fumigatus* | 2.150 | *A. fumigatus* | 2.099 |
| DTO 310-G5 | *A. neoniger* | No peaks found | 0 | No peaks found | 0 | No peaks found | 0 | No peaks found | 0 | No peaks found | 0 | No peaks found | 0 | No peaks found | 0 | No peaks found | 0 |
|  |  | *A. tubingensis* | 2.520 | *A. tubingensis* | 2.458 | *A. tubingensis* | 2.458 | *A. tubingensis* | 2.360 | *A. tubingensis* | 2.520 | *A. tubingensis* | 2.451 | *A. tubingensis* | 2.456 | *A. tubingensis* | 2.378 |
| DTO 310-G9 | *A. neoniger* | *A. tubingensis* | 2.646 | *A. tubigensis* | 2.620 | *A. tubingensis* | 2.597 | *A. tubingensis* | 2.570 | *A. tubingensis* | 2.646 | *A. tubigens* | 2.620 | *A. tubingensis* | 2.536 | *A. tubingensis* | 2.515 |
|  |  | *A. tubingensis* | 2.674 | *A. tubigensis* | 2.653 | *A. tubingensis* | 2.562 | *A. tubingensis* | 2.526 | *A. tubingensis* | 2.674 | *A. tubigens* | 2.653 | *A. tubingensis* | 2.543 | *A. tubingensis* | 2.542 |
| DTO 310-H1 | *A. flavus* | *A. flavus* | 2.427 | *A. flavus* | 2.395 | *A. flavus* | 2.343 | *A. flavus* | 2.330 | *A. flavus* | 2.395 | *A. flavus* | 2.314 | *A. flavus* | 2.427 | *A. flavus* | 2.382 |
|  |  | *A. flavus* | 2.516 | *A. flavus* | 2.485 | *A. flavus* | 2.365 | *A. flavus* | 2.316 | *A. flavus* | 2.462 | *A. flavus* | 2.457 | *A. flavus* | 2.516 | *A. flavus* | 2.485 |
| DTO 310-H3 | *A. flavus* | *A. flavus* | 2.339 | *A. flavus* | 2.282 | *A. flavus* | 2.166 | *A. oryzae* | 2.166 | *A. minisclerotigenes* | 2.211 | *A. flavus* | 2.160 | *A. flavus* | 2.339 | *A. flavus* | 2.282 |
|  |  | *A. flavus* | 2.680 | *A. flavus* | 2.614 | *A. flavus* | 2.385 | *A. flavus* | 2.367 | *A. flavus* | 2.602 | *A. flavus* | 2.602 | *A. flavus* | 2.680 | *A. flavus* | 2.614 |
| DTO 310-H4 | *A. flavus* | *A. flavus* | 2.508 | *A. flavus* | 2.467 | *A. oryzae* | 2.334 | *A. flavus* | 2.255 | *A. flavus* | 2.508 | *A. flavus* | 2.458 | *A. flavus* | 2.467 | *A. flavus* | 2.407 |
|  |  | *A. flavus* | 2.461 | *A. flavus* | 2.437 | *A. oryzae* | 2.197 | *A. austwickii* | 2.186 | *A. flavus* | 2.461 | *A. flavus* | 2.437 | *A. flavus* | 2.416 | *A. flavus* | 2.389 |
| DTO 310-H6 | *A. tamarii* | *A. tamarii* | 2.474 | *A. tamarii* | 2.419 | *A. pipericola* | 2.007 | *A. tamarii* | 1.998 | *A. tamarii* | 2.419 | *A. tamarii* | 2.419 | *A. tamarii* | 2.474 | *A. tamarii* | 2.310 |
|  |  | No peaks found | 0 | No peaks found | 0 | No peaks found | 0 | No peaks found | 0 | No peaks found | 0 | No peaks found | 0 | No peaks found | 0 | No peaks found | 0 |
| DTO 310-H7 | *A. calidoustus* | *A. ustus* | 2.388 | *A. ustus* | 1.771 | *A. ustus* | 2.388 | *A. ustus* | 1.771 | No reliable identification | 1.562 | No reliable identification | 1.399 | No reliable identification | 1.507 | No reliable identification | 1.352 |
|  |  | *A. ustus* | 2.494 | *A. ustus* | 1.847 | *A. ustus* | 2.494 | *A. ustus* | 1.847 | No reliable identification | 1.403 | No reliable identification | 1.398 | No reliable identification | 1.361 | No reliable identification | 1.233 |
| DTO 310-H8 | *A. fumigatus* | *A. fumigatus* | 1.764 | *A. fumigatus* | 1.745 | *A. fumigatus* | 1.764 | *A. fumigatus* | 1.745 | *A. fumigatu* | 1.745 | *No reliable identification* | 1.687 | No reliable identification | 1.550 | No reliable identification | 1.423 |
|  |  | *A. fumigatus* | 2.270 | *A. fumigatus* | 2.245 | *A. fumigatus* | 2.245 | *A. fumigatus* | 2.236 | *A. fumigatu* | 2.270 | *A. fumigatu* | 2.160 | *A. fumigatus* | 2.192 | *A. fumigatus* | 2.136 |
| DTO 310-I1 | *A. flavus* | *A. flavus* | 2.552 | *A. pipericola* | 2.498 | *A. oryzae* | 2.342 | *A. novoparasiticus* | 2.272 | *A. flavus* | 2.552 | *A. pipericola* | 2.498 | *A. flavus* | 2.474 | *A. aflatoxiformans* | 2.442 |
|  |  | No peaks found | 0 | No peaks found | 0 | No peaks found | 0 | No peaks found | 0 | No peaks found | 0 | No peaks found | 0 | No peaks found | 0 | No peaks found | 0 |
| DTO 310-I3 | *A. flavus* | *A. flavus* | 2.614 | *A. pipericola* | 2.608 | *A. flavus* | 2.377 | *A. novoparasiticus* | 2.341 | *A. flavus* | 2.614 | *A. pipericola* | 2.608 | *A. flavus* | 2.540 | *A. aflatoxiformans* | 2.536 |
|  |  | *A. flavus* | 2.566 | *A. pipericola* | 2.551 | *A. austwickii* | 2.345 | *A. flavus* | 2.326 | *A. flavus* | 2.566 | *A. pipericola* | 2.551 | *A. flavus* | 2.519 | *A. aflatoxiformans* | 2.504 |
| DTO 310-I4 | *A. tamarii* | *A. tamarii* | 2.206 | *A. tamarii* | 2.141 | *A. flavus* | 1.959 | *A. novoparasiticus* | 1.928 | *A. tamarii* | 2.141 | *A. flavus* | 2.061 | *A. tamarii* | 2.206 | *A. flavus* | 2.057 |
|  |  | *A. tamarii* | 2.426 | *A. tamarii* | 2.354 | *A. lanosus* | 2.038 | *A. austwickii* | 2.035 | *A. tamarii* | 2.354 | *A. pipericola* | 2.191 | *A. tamarii* | 2.426 | *A. cerealis* | 2.126 |
| DTO 310-I5 | *A. tamarii* | *A. tamarii* | 2.427 | *A. tamarii* | 2.402 | *A. pipericola* | 2.030 | *A. tamarii* | 1.975 | *A. tamarii* | 2.427 | *A. tamarii* | 2.370 | *A. tamarii* | 2.402 | *A. tamarii* | 2.142 |
|  |  | *A. tamarii* | 2.393 | *A. tamarii* | 2.387 | *A. togoensis* | 2.023 | *A. cerealis* | 1.959 | *A. tamarii* | 2.393 | *A. tamarii* | 2.313 | *A. tamarii* | 2.387 | *A. tamarii* | 2.198 |
| DTO 311-A3 | *A. tamarii* | *A. tamarii* | 2.457 | *A. tamarii* | 2.453 | *A. novoparasiticus* | 2.107 | *A. novoparasiticus* | 2.097 | *A. tamarii* | 2.453 | *A. pipericola* | 2.205 | *A. tamarii* | 2.457 | *A. tamarii* | 2.251 |
|  |  | *A. tamarii* | 2.453 | *A. tamarii* | 2.276 | *A. tamarii* | 1.952 | *A. tamarii* | 1.946 | *A. tamarii* | 2.276 | *A. pipericola* | 2.258 | *A. tamarii* | 2.453 | *A. tamarii* | 2.276 |
| DTO 311-B1 | *A. calidoustus* | *A. ustus* | 1.987 | No reliable identification | 1.314 | *A. ustus* | 1.987 | No reliable identification | 1.244 | No reliable identification | 1.295 | No reliable identification | 1.243 | No reliable identification | 1.314 | No reliable identification | 1.265 |
|  |  | *A. ustus* | 2.463 | *A. ustus* | 1.746 | *A. ustus* | 2.463 | *A. ustus* | 1.746 | No reliable identification | 1.544 | No reliable identification | 1.461 | No reliable identification | 1.432 | No reliable identification | 1.341 |
| DTO 311-B3 | *P. rubens* | *P. chrysogenum* | 2.221 | *P. chrysogenum* | 2.102 | *P. chrysogenum* | 2.221 | *P. chrysogenum* | 2.102 | No reliable identification | 1.212 | No reliable identification | 1.095 | No reliable identification | 1.203 | No reliable identification | 1.070 |
|  |  | *P. chrysogenum* | 2.338 | *P. chrysogenum* | 2.193 | *P. chrysogenum* | 2.338 | *P. chrysogenum* | 2.193 | No reliable identification | 1.054 | No reliable identification | 1.033 | No reliable identification | 1.010 | No reliable identification | 1.003 |
| DTO 311-B5 | *A. nomiae* | *A. nomiae* | 2.626 | *A. nomiae* | 2.566 | *A. nomiae* | 2.495 | *A. nomiae* | 2.369 | *A. nomiae* | 2.626 | *A. nomiae* | 2.566 | *A. nomiae* | 2.451 | *A. nomiae* | 2.442 |
|  |  | *A. nomiae* | 2.571 | *A. nomiae* | 2.472 | *A. nomiae* | 2.463 | *A. nomiae* | 2.356 | *A. nomiae* | 2.571 | *A. nomiae* | 2.471 | *A. nomiae* | 2.472 | *A. nomiae* | 2.424 |
| DTO 311-B7 | *P. rubens* | *P. chrysogenum* | 2.147 | *P. chrysogenum* | 2.075 | *P. chrysogenum* | 2.147 | *P. chrysogenum* | 2.075 | No reliable identification | 1.011 | No reliable identification | 0.916 | No reliable identification | 1.138 | No reliable identification | 0.969 |
|  |  | *P. chrysogenum* | 2.243 | *P. chrysogenum* | 2.047 | *P. chrysogenum* | 2.243 | *P. chrysogenum* | 2.047 | No reliable identification | 1.159 | No reliable identification | 1.088 | No reliable identification | 1.055 | No reliable identification | 0.977 |
| DTO 311-B8 | *A. neoniger* | *A. tubingensis* | 2.696 | *A. tubigensis* | 2.683 | *A. tubingensis* | 2.650 | *A. tubingensis* | 2.557 | *A. tubingensis* | 2.696 | *A. tubingensis* | 2.683 | *A. tubingensis* | 2.649 | *A. tubingensis* | 2.600 |
|  |  | *A. tubingensis* | 2.665 | *A. tubingensis* | 2.651 | *A. tubingensis* | 2.625 | *A. tubingensis* | 2.463 | *A. tubingensis* | 2.665 | *A. tubingensis* | 2.614 | *A. tubingensis* | 2.651 | *A. tubingensis* | 2.596 |
| DTO 311-C3 | *A. flavus* | *A. flavus* | 2.197 | *A. flavus* | 2.187 | *A. aflatoxiformans* | 2.153 | *A. cerealis* | 2.140 | *A. flavus* | 2.184 | *A. flavus* | 2.135 | *A. flavus* | 2.197 | *A. flavus* | 2.187 |
|  |  | *A. flavus* | 2.587 | *A. flavus* | 2.538 | *A. austwickii* | 2.407 | *A. aflatoxiformans* | 2.385 | *A. flavus* | 2.587 | *A. flavus* | 2.515 | *A. flavus* | 2.538 | *A. flavus* | 2.460 |
| DTO 311-C5 | *P. rubens* | *P. chrysogenum* | 2.016 | *P. chrysogenum* | 1.982 | *P. chrysogenum* | 2.016 | *P. chrysogenum* | 1.982 | No reliable identification | 1.092 | No reliable identification | 0.985 | No reliable identification | 1.067 | No reliable identification | 1.034 |
|  |  | *P. chrysogenum* | 2.149 | *P. chrysogenum* | 2.138 | *P. chrysogenum* | 2.149 | *P. chrysogenum* | 2.138 | No reliable identification | 1.023 | No reliable identification | 1.007 | No reliable identification | 1.020 | No reliable identification | 1.011 |
| DTO 311-C9 | *A. fumigatus* | *A. fumigatus* | 2.337 | *A. fumigatus* | 2.326 | *A. fumigatus* | 2.337 | *A. fumigatus* | 2.326 | *A. fumigatus* | 2.202 | *A. fumigatus* | 2.177 | *A. fumigatus* | 2.209 | *A. fumigatus* | 2.129 |
|  |  | *A. fumigatus* | 2.481 | *A. fumigatus* | 2.416 | *A. fumigatus* | 2.481 | *A. fumigatus* | 2.416 | *A. fumigatus* | 2.325 | *A. fumigatus* | 2.323 | *A. fumigatus* | 2.344 | *A. fumigatus* | 2.273 |
| DTO 311-D1 | *A. fumigatus* | *A. fumigatus* | 2.415 | *A. fumigatus* | 2.395 | *A. fumigatus* | 2.415 | *A. fumigatus* | 2.395 | *A. fumigatus* | 2.266 | *A. fumigatus* | 2.252 | *A. fumigatus* | 2.260 | *A. fumigatus* | 2.146 |
|  |  | *A. fumigatus* | 2.411 | *A. fumigatus* | 2.357 | *A. fumigatus* | 2.411 | *A. fumigatus* | 2.357 | *A. fumigatus* | 2.252 | *A. fumigatus* | 2.168 | *A. fumigatus* | 2.282 | *A. fumigatus* | 2.210 |
| DTO 311-D4 | *A. nomiae* | *A. nomiae* | 2.545 | *A. nomiae* | 2.528 | *A. nomiae* | 2.337 | *A. nomiae* | 2.272 | *A. nomiae* | 2.545 | *A. nomiae* | 2.528 | *A. nomiae* | 2.207 | *A. nomiae* | 2.157 |
|  |  | *A. nomiae* | 2.662 | *A. nomiae* | 2.658 | *A. nomiae* | 2.448 | *A. nomiae* | 2.326 | *A. nomiae* | 2.662 | *A. nomiae* | 2.658 | *A. nomiae* | 2.236 | *A. nomiae* | 2.221 |
| DTO 311-F9 | *A. flavus* | No peaks found | 0 | No peaks found | 0 | No peaks found | 0 | No peaks found | 0 | No peaks found | 0 | No peaks found | 0 | No peaks found | 0 | No peaks found | 0 |
|  |  | *A. flavus* | 2.584 | *A. pipericola* | 2.559 | *A. flavus* | 2.327 | *A. oryzae* | 2.302 | *A. flavus* | 2.584 | *A. pipericola* | 2.559 | *A. aflatoxiformans* | 2.534 | *A. flavus* | 2.477 |
| DTO 311-G1 | *A. tamarii* | *A. tamarii* | 2.525 | *A. tamarii* | 2.461 | *A. tamarii* | 2.033 | *A. flavus* | 2.021 | *A. tamarii* | 2.461 | *A. tamarii* | 2.457 | *A. tamarii* | 2.525 | *A. tamarii* | 2.360 |
|  |  | *A. tamarii* | 2.453 | *A. tamarii* | 2.442 | *A. flavus* | 2.038 | *A. pipericola* | 2.017 | *A. tamarii* | 2.442 | *A. tamarii* | 2.431 | *A. tamarii* | 2.453 | *A. tamarii* | 2.345 |
| DTO 311-G2 | *A. fumigatus* | *A. fumigatus* | 2.260 | *A. fumigatus* | 2.254 | *A. fumigatus* | 2.260 | *A. fumigatus* | 2.254 | *A. fumigatus* | 2.136 | *A. fumigatus* | 2.124 | *A. fumigatus* | 2.238 | *A. fumigatus* | 2.207 |
|  |  | *A. fumigatus* | 1.872 | *A. fumigatus* | 1.754 | *A. fumigatus* | 1.872 | *A. fumigatus* | 1.754 | No reliable identification | 1.686 | No reliable identifi | 1.557 | *A. fumigatus* | 1.743 | No reliable identification | 1.475 |

1PC: Growth on top of a polycarbonate filter. 2PC: Growth between two polycarbonate filters. LC: Liquid cultivation.

**Suppl. Table X: MALDI-TOF MS identification results of Qatar clinical *Aspergillus* isolates.**

| **Strain no.** | **Molecular ID** | **Identification** | **Score** | **Identification** | **Score** |
| --- | --- | --- | --- | --- | --- |
|  |  | **(best match)** |  | **(second best match)** |  |
| 486 | *Aspergillus caespitosus* | *Aspergillus caespitosus* | 2.480 | *Aspergillus caespitosus* | 2.420 |
| 6630 | *Aspergillus chevalieri* | *Aspergillus chevalieri* | 2.480 | *Aspergillus chevalieri* | 2.390 |
| 1787 | *Aspergillus citrinoterreus* | *Aspergillus terreus* | 2.335 | *Aspergillus terreus* | 2.316 |
| 5260 | *Aspergillus citrinoterreus* | *Aspergillus terreus* | 2.410 | *Aspergillus terreus* | 2.275 |
| 6070 | *Aspergillus citrinoterreus* | *Aspergillus terreus* | 2.399 | *Aspergillus terreus* | 2.398 |
| 234-B | *Aspergillus flavus* | *Aspergillus flavus* | 2.697 | *Aspergillus flavus* | 2.683 |
| 98 | *Aspergillus flavus* | *Aspergillus flavus* | 2.513 | *Aspergillus flavus* | 2.469 |
| 139 | *Aspergillus flavus* | *Aspergillus flavus* | 2.381 | *Aspergillus flavus* | 2.375 |
| 180 | *Aspergillus flavus* | *Aspergillus flavus* | 2.651 | *Aspergillus flavus* | 2.606 |
| 224 | *Aspergillus flavus* | *Aspergillus flavus* | 2.682 | *Aspergillus flavus* | 2.663 |
| 338 | *Aspergillus flavus* | *Aspergillus flavus* | 2.591 | *Aspergillus aflatoxiformans* | 2.526 |
|  |  | *Aspergillus flavus* | 2.595 | *Aspergillus flavus* | 2.562 |
| 518 | *Aspergillus flavus* | *Aspergillus flavus* | 2.632 | *Aspergillus flavus* | 2.565 |
|  |  | *Aspergillus flavus* | 2.639 | *Aspergillus aflatoxiformans* | 2.519 |
| 725 | *Aspergillus flavus* | *Aspergillus aflatoxiformans* | 2.385 | *Aspergillus flavus* | 2.365 |
|  |  | *Aspergillus aflatoxiformans* | 2.369 | *Aspergillus pipericola* | 2.341 |
| 861 | *Aspergillus flavus* | *Aspergillus flavus* | 2.596 | *Aspergillus flavus* | 2.520 |
| 878 | *Aspergillus flavus* | *Aspergillus flavus* | 2.648 | *Aspergillus flavus* | 2.620 |
| 1013 | *Aspergillus flavus* | *Aspergillus flavus* | 2.602 | *Aspergillus flavus* | 2.579 |
| 1129 | *Aspergillus flavus* | *Aspergillus flavus* | 2.656 | *Aspergillus flavus* | 2.627 |
| 1165 | *Aspergillus flavus* | *Aspergillus aflatoxiformans* | 2.457 | *Aspergillus aflatoxiformans* | 2.437 |
|  |  | *Aspergillus flavus* | 2.544 | *Aspergillus aflatoxiformans* | 2.484 |
| 1169 | *Aspergillus flavus* | *Aspergillus aflatoxiformans* | 2.478 | *Aspergillus flavus* | 2.475 |
|  |  | *Aspergillus flavus* | 2.517 | *Aspergillus aflatoxiformans* | 2.485 |
| 1374 | *Aspergillus flavus* | *Aspergillus aflatoxiformans* | 2.538 | *Aspergillus aflatoxiformans* | 2.500 |
|  |  | *Aspergillus flavus* | 2.646 | *Aspergillus flavus* | 2.624 |
| 3118 | *Aspergillus flavus* | *Aspergillus flavus* | 2.629 | *Aspergillus flavus* | 2.578 |
| 3252 | *Aspergillus flavus* | *Aspergillus flavus* | 2.684 | *Aspergillus flavus* | 2.634 |
| 5254 | *Aspergillus flavus* | *Aspergillus flavus* | 2.545 | *Aspergillus aflatoxiformans* | 2.535 |
| 6198 | *Aspergillus flavus* | *Aspergillus aflatoxiformans* | 2.541 | *Aspergillus aflatoxiformans* | 2.530 |
|  |  | *Aspergillus aflatoxiformans* | 2.499 | *Aspergillus aflatoxiformans* | 2.492 |
| 120 | *Aspergillus fumigatus* | *Aspergillus fumigatus* | 2.390 | *Aspergillus fumigatus* | 2.322 |
| 567 | *Aspergillus fumigatus* | *Aspergillus fumigatus* | 2.351 | *Aspergillus fumigatus* | 2.335 |
| 688 | *Aspergillus fumigatus* | *Aspergillus fumigatus* | 2.318 | *Aspergillus fumigatus* | 2.162 |
| 700 | *Aspergillus fumigatus* | *Aspergillus fumigatus* | 2.406 | *Aspergillus fumigatus* | 2.314 |
| 807 | *Aspergillus fumigatus* | *Aspergillus fumigatus* | 2.396 | *Aspergillus fumigatus* | 2.372 |
| 1047 | *Aspergillus fumigatus* | *Aspergillus fumigatus* | 2.058 | *Aspergillus fumigatus* | 1.927 |
| 1177 | *Aspergillus fumigatus* | *Aspergillus fumigatus* | 2.312 | *Aspergillus fumigatus* | 2.253 |
| 1332 | *Aspergillus fumigatus* | *Aspergillus fumigatus* | 2.323 | *Aspergillus fumigatus* | 2.250 |
| 1490 | *Aspergillus fumigatus* | *Aspergillus fumigatus* | 2.410 | *Aspergillus fumigatus* | 2.294 |
| 6057 | *Aspergillus fumigatus* | *Aspergillus fumigatus* | 2.324 | *Aspergillus fumigatus* | 2.298 |
| 7675 | *Aspergillus fumigatus* | *Aspergillus fumigatus* | 2.483 | *Aspergillus fumigatus* | 2.398 |
| 1000609 | *Aspergillus fumigatus* | *Aspergillus fumigatus* | 2.311 | *Aspergillus fumigatus* | 2.269 |
| 140 | *Aspergillus nidulans* | *Aspergillus nidulans* | 2.350 | *Aspergillus nidulans* | 2.331 |
| 205 | *Aspergillus nidulans* | *Aspergillus nidulans* | 2.303 | *Aspergillus nidulans* | 2.303 |
| 477 | *Aspergillus pallidofulvus* | *Aspergillus pallidofulvus* | 2.530 | *Aspergillus ochraceus* | 2.180 |
| 782 | *Aspergillus pseudonomiae* | *Aspergillus pseudonomiae* | 2.638 | *Aspergillus pseudonomiae* | 2.499 |
| 1301 | *Aspergillus quadrilineatus* | *Aspergillus nidulans* | 2.179 | *Aspergillus nidulans* | 2.155 |
| 800006 | *Aspergillus sublatus* | *Aspergillus nidulans* | 2.574 | *Aspergillus nidulans* | 2.431 |
| 521 | *Aspergillus tamarii* | *Aspergillus tamarii* | 2.238 | *Aspergillus tamarii* | 2.235 |
| 78 | *Aspergillus terreus* | *Aspergillus terreus* | 2.493 | *Aspergillus terreus* | 2.427 |
| 334 | *Aspergillus terreus* | *Aspergillus terreus* | 2.331 | *Aspergillus terreus* | 2.326 |
| 438 | *Aspergillus terreus* | *Aspergillus terreus* | 2.247 | *Aspergillus terreus* | 2.215 |
| 1444 | *Aspergillus terreus* | *Aspergillus terreus* | 2.562 | *Aspergillus terreus* | 2.489 |
| 1467 | *Aspergillus terreus* | *Aspergillus terreus* | 2.256 | *Aspergillus terreus* | 2.230 |
| 1651 | *Aspergillus terreus* | *Aspergillus terreus* | 2.246 | *Aspergillus terreus* | 2.191 |
| 2779 | *Aspergillus terreus* | *Aspergillus terreus* | 2.260 | *Aspergillus terreus* | 2.208 |
| 3996 | *Aspergillus terreus* | *Aspergillus terreus* | 2.433 | *Aspergillus terreus* | 2.411 |
| 4145 | *Aspergillus terreus* | *Aspergillus terreus* | 2.387 | *Aspergillus terreus* | 2.304 |
| 4260 | *Aspergillus terreus* | *Aspergillus terreus* | 2.453 | *Aspergillus terreus* | 2.405 |
| 4672 | *Aspergillus terreus* | *Aspergillus terreus* | 2.464 | *Aspergillus terreus* | 2.457 |
| 6596 | *Aspergillus terreus* | *Aspergillus terreus* | 2.354 | *Aspergillus terreus* | 2.295 |
| 6746 | *Aspergillus terreus* | *Aspergillus terreus* | 2.441 | *Aspergillus terreus* | 2.423 |
| 6811 | *Aspergillus terreus* | *Aspergillus terreus* | 2.333 | *Aspergillus terreus* | 2.296 |
| 7406 | *Aspergillus terreus* | *Aspergillus terreus* | 2.356 | *Aspergillus terreus* | 2.352 |
| 7463 | *Aspergillus terreus* | *Aspergillus terreus* | 2.315 | *Aspergillus terreus* | 2.248 |
| 4000006 | *Aspergillus terreus* | *Aspergillus terreus* | 2.319 | *Aspergillus terreus* | 2.318 |
| 12 | *Aspergillus tubingensis* | *Aspergillus tubigensis* | 2.539 | *Aspergillus tubingensis* | 2.526 |
| 333 | *Aspergillus tubingensis* | *Aspergillus tubingensis* | 2.696 | *Aspergillus tubingensis* | 2.591 |
| 1072 | *Aspergillus tubingensis* | *Aspergillus tubingensis* | 2.605 | *Aspergillus tubingensis* | 2.601 |
| 234-A | *Aspergillus welwitschiae* | *Aspergillus niger* | 2.703 | *Aspergillus niger* | 2.695 |
| 404 | *Aspergillus welwitschiae* | *Aspergillus niger* | 2.450 | *Aspergillus niger* | 2.410 |
| 416 | *Aspergillus welwitschiae* | *Aspergillus niger* | 2.159 | *Aspergillus niger* | 2.051 |
| 490 | *Aspergillus welwitschiae* | *Aspergillus niger* | 2.512 | *Aspergillus niger* | 2.410 |
| 676 | *Aspergillus welwitschiae* | *Aspergillus niger* | 2.693 | *Aspergillus niger* | 2.625 |
| 1114 | *Aspergillus welwitschiae* | *Aspergillus niger* | 2.439 | *Aspergillus niger* | 2.435 |
| 2266 | *Aspergillus welwitschiae* | *Aspergillus niger* | 2.532 | *Aspergillus niger* | 2.485 |

**Suppl. Table XI: Identification errors for the Qatar clinical isolates.**

| **Isolate no.** | **Molecular ID** | **Identification by MALDI-TOF MS** | | | | **Error (minor/major)** |
| --- | --- | --- | --- | --- | --- | --- |
|  |  | **1^st^ best match** | **Score** | **2^nd^ best match** | **Score** |  |
| 1787 | *Aspergillus citrinoterreus* | *Aspergillus terreus* | 2.335 | *Aspergillus terreus* | 2.316 | Minor |
| 5260 | *Aspergillus citrinoterreus* | *Aspergillus terreus* | 2.410 | *Aspergillus terreus* | 2.275 | Minor |
| 6070 | *Aspergillus citrinoterreus* | *Aspergillus terreus* | 2.399 | *Aspergillus terreus* | 2.398 | Minor |
| 1374 | *Aspergillus flavus* | *Aspergillus aflatoxiformans* | 2.538 | *Aspergillus aflatoxiformans* | 2.500 | Minor |
|  |  | *Aspergillus flavus* | 2.646 | *Aspergillus flavus* | 2.624 | Minor |
| 725 | *Aspergillus flavus* | *Aspergillus aflatoxiformans* | 2.385 | *Aspergillus flavus* | 2.365 | Minor |
|  |  | *Aspergillus aflatoxiformans* | 2.369 | *Aspergillus pipericola* | 2.341 | Minor |
| 1169 | *Aspergillus flavus* | *Aspergillus aflatoxiformans* | 2.478 | *Aspergillus flavus* | 2.475 | Minor |
|  |  | *Aspergillus flavus* | 2.517 | *Aspergillus aflatoxiformans* | 2.485 | Minor |
| 1165 | *Aspergillus flavus* | *Aspergillus aflatoxiformans* | 2.457 | *Aspergillus aflatoxiformans* | 2.437 | Minor |
|  |  | *Aspergillus flavus* | 2.544 | *Aspergillus aflatoxiformans* | 2.484 | Minor |
| 6198 | *Aspergillus flavus* | *Aspergillus aflatoxiformans* | 2.541 | *Aspergillus aflatoxiformans* | 2.530 | Minor |
| 1301 | *Aspergillus quadrilineatus* | *Aspergillus nidulans* | 2.179 | *Aspergillus nidulans* | 2.155 | Minor |
| 800006 | *Aspergillus sublatus* | *Aspergillus nidulans* | 2.574 | *Aspergillus nidulans* | 2.431 | Minor |
| 234-A | *Aspergillus welwitschiae* | *Aspergillus niger* | 2.703 | *Aspergillus niger* | 2.695 | Minor |
| 404 | *Aspergillus welwitschiae* | *Aspergillus niger* | 2.450 | *Aspergillus niger* | 2.410 | Minor |
| 416 | *Aspergillus welwitschiae* | *Aspergillus niger* | 2.159 | *Aspergillus niger* | 2.051 | Minor |
| 490 | *Aspergillus welwitschiae* | *Aspergillus niger* | 2.512 | *Aspergillus niger* | 2.410 | Minor |
| 676 | *Aspergillus welwitschiae* | *Aspergillus niger* | 2.693 | *Aspergillus niger* | 2.625 | Minor |
| 1114 | *Aspergillus welwitschiae* | *Aspergillus niger* | 2.439 | *Aspergillus niger* | 2.435 | Minor |
| 2266 | *Aspergillus welwitschiae* | *Aspergillus niger* | 2.532 | *Aspergillus niger* | 2.485 | Minor |
